# A nomogram predictive model for long-term survival in spontaneous intracerebral hemorrhage patients without cerebral herniation at admission

**DOI:** 10.1038/s41598-022-26176-0

**Published:** 2023-02-22

**Authors:** Fuxin Lin, Qiu He, Lingyun Zhuo, Mingpei Zhao, Gengzhao Ye, Zhuyu Gao, Wei Huang, Lveming Cai, Fangyu Wang, Huangcheng Shangguan, Wenhua Fang, Yuanxiang Lin, Dengliang Wang, Dezhi Kang

**Affiliations:** 1 Department of Neurosurgery, Neurosurgery Research Institute, The First Affiliated Hospital of Fujian Medical University, Fuzhou, Fujian China; 2 Clinical Research and Translation Center, The First Affiliated Hospital of Fujian Medical University, Fuzhou, Fujian China; 3 Fujian Clinical Research Center for Neurological Diseases, The First Affiliated Hospital of Fujian Medical University, Fuzhou, Fujian China; 4 Fujian Provincial Institutes of Brain Disorders and Brain Sciences, First Affiliated Hospital, Fujian Medical University, Fuzhou, 350005 Fujian China

**Keywords:** Neurology, Risk factors

## Abstract

Stratification of spontaneous intracerebral hemorrhage (sICH) patients without cerebral herniation at admission, to determine the subgroups may be suffered from poor outcomes or benefit from surgery, is important for following treatment decision. The aim of this study was to establish and verify a de novo nomogram predictive model for long-term survival in sICH patients without cerebral herniation at admission. This study recruited sICH patients from our prospectively maintained ICH patient database (RIS-MIS-ICH, ClinicalTrials.gov Identifier: NCT03862729) between January 2015 and October 2019. All eligible patients were randomly classified into a training cohort and a validation cohort according to the ratio of 7:3. The baseline variables and long-term survival outcomes were collected. And the long-term survival information of all the enrolled sICH patients, including the occurrence of death and overall survival. Follow-up time was defined as the time from the onset to death of the patient or the last clinical visit. The nomogram predictive model was established based on the independent risk factors at admission for long-term survival after hemorrhage. The concordance index (C-index) and ROC curve were used to evaluate the accuracy of the predictive model. Discrimination and calibration were used to validate the nomogram in both the training cohort and the validation cohort. A total of 692 eligible sICH patients were enrolled. During the average follow-up time of 41.77 ± 0.85 months, a total of 178 (25.7%) patients died. The Cox Proportional Hazard Models showed that age (HR 1.055, 95% CI 1.038–1.071, *P* < 0.001), Glasgow Coma Scale (GCS) at admission (HR 2.496, 95% CI 2.014–3.093, *P* < 0.001) and hydrocephalus caused by intraventricular hemorrhage (IVH) (HR 1.955, 95% CI 1.362–2.806, *P* < 0.001) were independent risk factors. The C index of the admission model was 0.76 and 0.78 in the training cohort and validation cohort, respectively. In the ROC analysis, the AUC was 0.80 (95% CI 0.75–0.85) in the training cohort and was 0.80 (95% CI 0.72–0.88) in the validation cohort. SICH patients with admission nomogram scores greater than 87.75 were at high risk of short survival time. For sICH patients without cerebral herniation at admission, our de novo nomogram model based on age, GCS and hydrocephalus on CT may be useful to stratify the long-term survival outcomes and provide suggestions for treatment decision-making.

## Introduction

Spontaneous intracerebral hemorrhage (sICH) is one of the leading causes of death and disability, and there are substantial economic costs for post-stroke care^1^. Although there have been some technological advances in surgery and rehabilitation, the long-term functional outcomes and mortality have not been improved significantly^2^. For patients with acute cerebral herniation, hematoma evacuation with decompressive craniectomy can be considered as a life-saving measure^3^. However, for patients without cerebral herniation, surgical options have been repeatedly evaluated in large multicenter randomized controlled trials, that unfortunately have not demonstrated improved outcomes^4–7^. Therefore, stratification of sICH patients without cerebral herniation at admission, to determine the subgroup may be suffered from poor outcomes or benefit from surgery, is important for following treatment decision. However, in the literature, almost all of prognostic prediction studies were derived from the general ICH population, and selected 30-day and 90-day (short-term) survival or functional deficits as outcome indicators^8–13^. As we know, sICH patients without cerebral herniation in the acute phase might have a longer survival period and better functional outcomes than general ICH population. To the best of our knowledge, the long-term survival data and predictive models are lack for this kind of patients in the literature. Therefore, the aim of this study was to review the long-term survival data in a large cohort, to determine the risk factors, to establish the nomogram predictive models, and to validate the predictive value of the nomogram predictive models at admission for sICH patients without acute cerebral herniation.

## Methods

### Patients

This study recruited sICH patients from our prospectively maintained ICH patient database (RIS-MIS-ICH, ClinicalTrials.gov Identifier: NCT03862729) between January 2015 and October 2019. The criteria for enrollment were as follows: (1) Emergent computed tomography (CT)/computed tomographic angiography (CTA) showed a sICH within 48 h from onset (patient with a small amount of IVH is eligible); (2) Without cerebral herniation at admission; (3) Patients were treated by observation before hemorrhage growth or symptoms deterioration; (5) Informed consent, and willing to accept long-term follow-up. The exclusion criteria were as follows: (1) ICH secondary to an underlying structural cause identified by brain imaging, (ie, vascular malformation, aneurysm, tumor, etc.); (2) Patients had a severe pre-existing physical or mental disability or severe comorbidity that might interfere with the assessment of survival outcome; (3) Patients had an infratentorial hemorrhage or extension of a supratentorial hemorrhage into the brainstem. Ethical approval was obtained through the relevant ethics committee of the First Affiliated Hospital of Fujian Medical University (Ethical Approval Number: MRCTA, ECFAH of FMU [2018] 082-1). And this study also follows the relevant Chinese laws, regulations and guidelines, as well as international laws and regulations.

### Variables

Data collection, data management and quality control can refer to the registration information of the RIS-MIS-ICH study on clinicaltrials.gov (NCT03862729). The project-related data was collected through the electronic data registry system (Real Data Medical Research Inc.). The radiological image information and follow-up data were determined by two neurosurgeons (Q H and DL W) with consensus.

The data retrieved from our database including baseline variables and long-term survival outcomes. The baseline variables were as follows: (1) Personal information; (2) History of present illness; (3) Physical examination at emergent department; (4) Laboratory assay at the emergent department; (5) Radiological imaging at the emergent department (initial CT/CTA): including hematoma location, depth of hematoma, whether with IVH or not, whether hydrocephalus occurred, hematoma volume, perihematomal edema volume, hematoma shape and density categorical scales score, black hole sign, island sign, swirl sign, blood-fluid level in hematoma. And the density categorical scales score includes 2 novel 5-point categorical scales were created, reflecting the spectrum of appearance of ICH shape and Hounsfield unit density variation, to provide an agreed visual representation of the terms “regular/irregular” and “homogeneous/heterogeneous^14^. The black hole sign was defined as hypoattenuatting area encapsulated within the hyperattenuating hematoma with a clearly defined border^15^. The island sign was defined as (1) ≥ 3 scattered small hematomas all separate from the main hematoma or (2) ≥ 4 small hematomas some or all of which may connect with the main hematoma. The scattered small hematomas (separate islands) could be round or oval and are separate from the main hematoma. The small hematomas that connect with the main hematoma (connected islands) should be bubble-like or sprout-like but not lobulated^16^. The swirl sign is recognized as an area of low attenuation within an extraaxial hyperattenuating fluid collection^17^. The blood-fluid level within the hematoma, defined as a change within the hematoma resulting in a linear interface between 2 discrete fluid densities^18,19^.

This study focused on the long-term survival information of all the enrolled sICH patients, including the occurrence of death and overall survival (OS). Follow-up time was defined as the time from the onset to death of the patient or the last clinical visit. During the follow-up period, all patients enrolled were regularly followed up at the outpatient department or by phone call. The patient’s death information was further confirmed in the local public security system to ensure the accuracy of the data. The follow-ups of all patients were conducted by trained and experienced clinicians (GZ Y, MP Z, ZY G, W H, LM C, FY W, HC SG).

### Statistical analysis

All patients enrolled were randomly divided into the training cohort and the validation cohort according to the ratio of 7:3. The training cohort was used to establish the predictive model, and the validation cohort was used to validate the results. The independent prognostic factors related to follow-up death were assessed through univariate analysis and multivariate Cox proportional hazard models. Variables with *P* values less than 0.05 in univariate analysis were considered as potentially related factors and included in the COX proportional hazard model for further analysis. The results were presented as hazard ratio (HR) with 95% Confidence intervals (CIs).

A nomogram predictive model for death probability after ictus was constructed based on the hazard factors at admission identified by the regression model. Discrimination and calibration, as two main aspects of the performance of a model, were used to validate the nomogram. Calibration curves were drawn to show the relationship between the actual probability and the predicted probability. Meanwhile, the clinical value of the model was assessed by decision curve analysis (DCA). In addition, the ROC curves were drawn and the AUC values and C-index were further calculated to evaluate the predictive accuracy of the nomogram model in the training cohort and validation cohort.

Risk scores for variables were obtained based on a nomogram, and a total risk score was calculated for each patient. The maximally selected rank statistics as implemented in the maxstat package was used to further determine the cutoff value of the total risk score to separate the population into high and low risk groups^20,21^. In order to further explore the influence of dichotomized risk in the final prediction model on death, survival analysis by Kaplan–Meier method was further carried out and Log-rank test was adopted to compare the survival curves of two groups.

Statistical description, univariate analysis, and Cox proportional hazards regression model were performed by SPSS Statistics software (version 25.0, SPSS Inc., Chicago, USA). Random grouping, drawing of nomogram, calibration plot, ROC curve and DCA curve, and C-index calculation were constructed by the relevant packages (i.e., caret, rms, Hmisc, ROCR, maxstat, and rmda) in R language software (version 4.1.0, Institute for Statistics and Mathematics, Vienna, Austria). Throughout the study, all tests were two-sided and *P* < 0.05 was defined as statistically significant.

### Ethical approval

Ethical approval was obtained through the relevant ethics committee of the First Affiliated Hospital of Fujian Medical University (Ethical Approval Number: MRCTA, ECFAH of FMU [2018] 082-1). The enrolled patients/participants provided their written informed consent to participate in this study.

## Results

### Study population and survival outcomes

**Figure 1:**
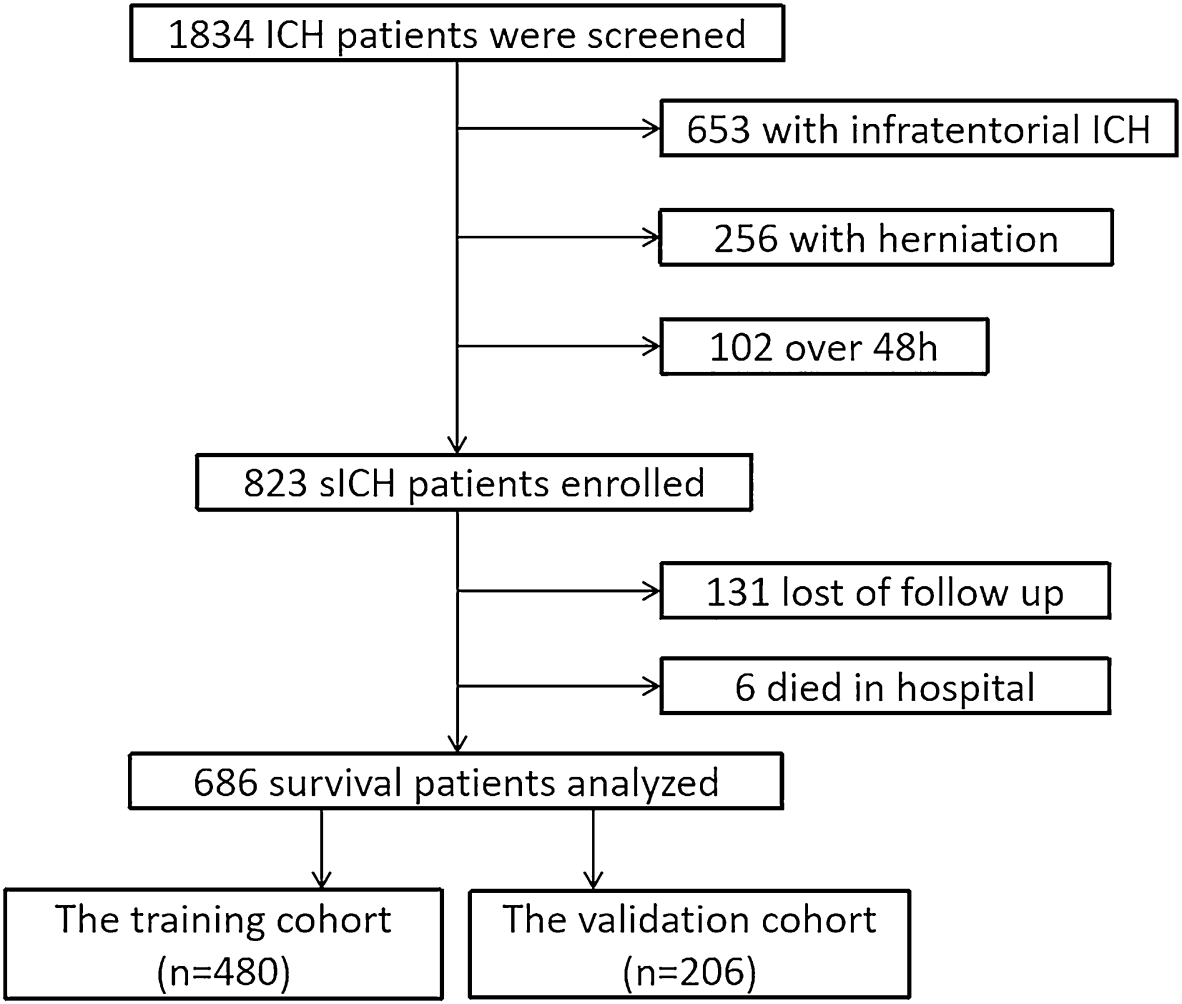
The screening flow chart of the study population.

A total of 1834 patients were screened in this study. Of them, 653 patients with infratentorial ICH, 256 patients with acute cerebral herniation, and 102 patients over 48 h from ictus were excluded. Overall, 823 patients met the study inclusion criteria, 131 of whom were lost to follow-up, and 692 were followed up for long-term survival data (Fig. 1). Patient demographics were shown in detail in Supplementary Table 1. Patients lost to follow-up had significantly lower breath rate at admission (57.35 ± 11.62 vs. 60.45 ± 12.32, *P* = 0.006), more supratentorial deep-seated hematoma (90.8% vs. 82.7%, *P* = 0.019), lower rate of IVH (7.6% vs. 14.6%), and less obstructive hydrocephalus in the fourth ventricle (3.1% vs. 8.2%).

**Figure 2:**
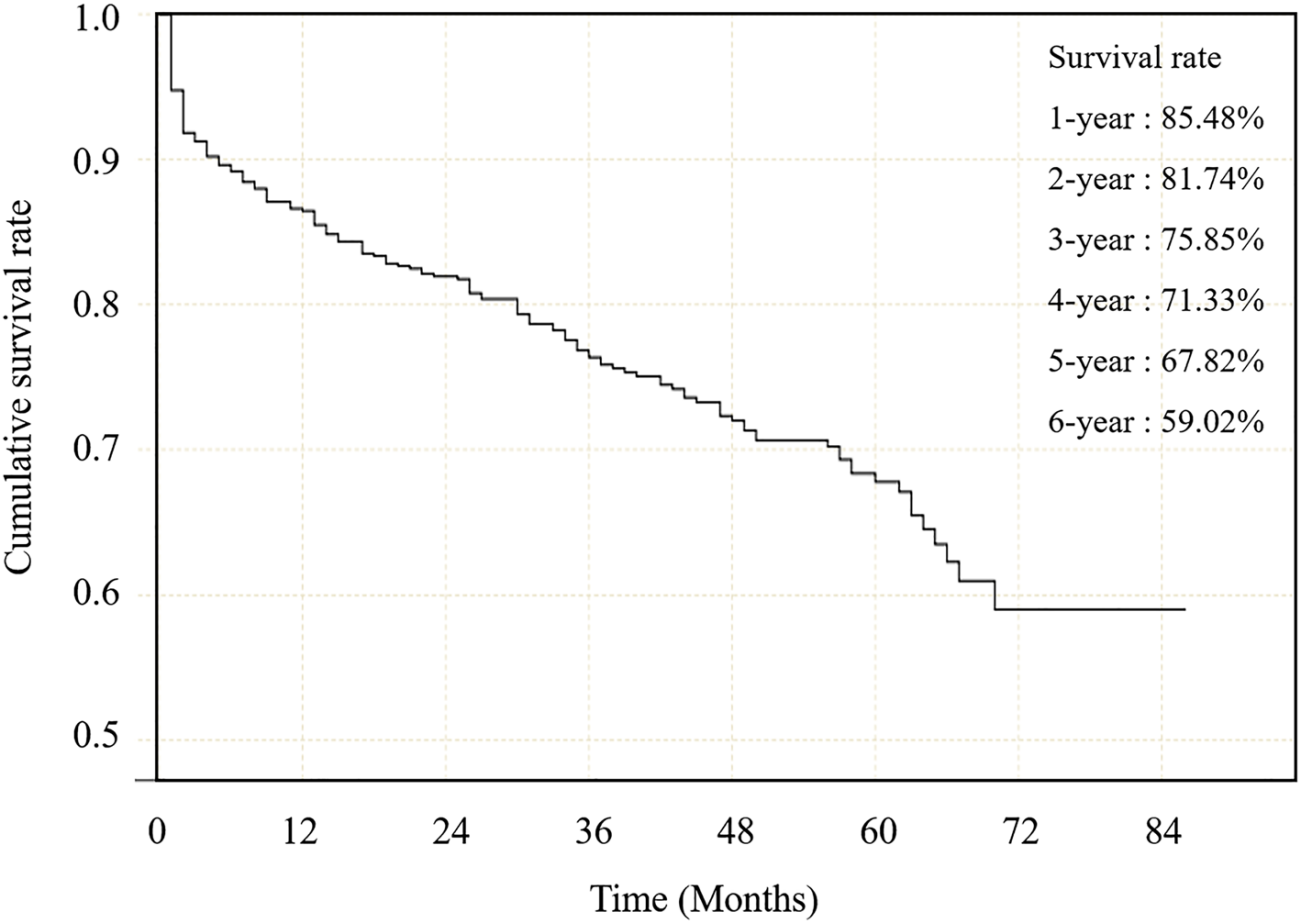
Survival curve of 692 sICH patients.

For the 692 sICH patients included in the analysis, there were twice as many males (70.5%) as females (29.5%), with a mean age of 60.45 ± 12.32 years old. The median time of onset-to-hospital interval was 12 (6–24) hours, and the median hematoma volume was 12.83 ml. Most of the patients were awake or with mild coma (GCS 13–15). A majority of the patients had deep-seated hematoma (82.7%), but only a part of them had IVH (36.3%). Most of the patients were treated by conservative treatment (73.8%) with an average length of hospital stay of 16 (12–21) days. With an average follow-up time of 41.77 ± 0.85 months, a total of 178 (25.7%) patients died. Survival rates of 1 to 6 years for sICH patients without acute herniation were 85.48%, 81.74%, 75.85%, 71.33%, 67.82%, and 59.02%, respectively (Fig. 2).

### Risk factors and predictive models

In the current study, 692 patients were randomly divided into the training cohort (n = 484) and the validation cohort (n = 208) at a ratio of 7:3. There were 128 (26.4%) and 50 (24.0%) deaths in the two groups, respectively. Except for the onset-to-hospital interval, the remaining variables were balanced and comparable in both datasets (Supplementary Table 2).

**Figure 3:**
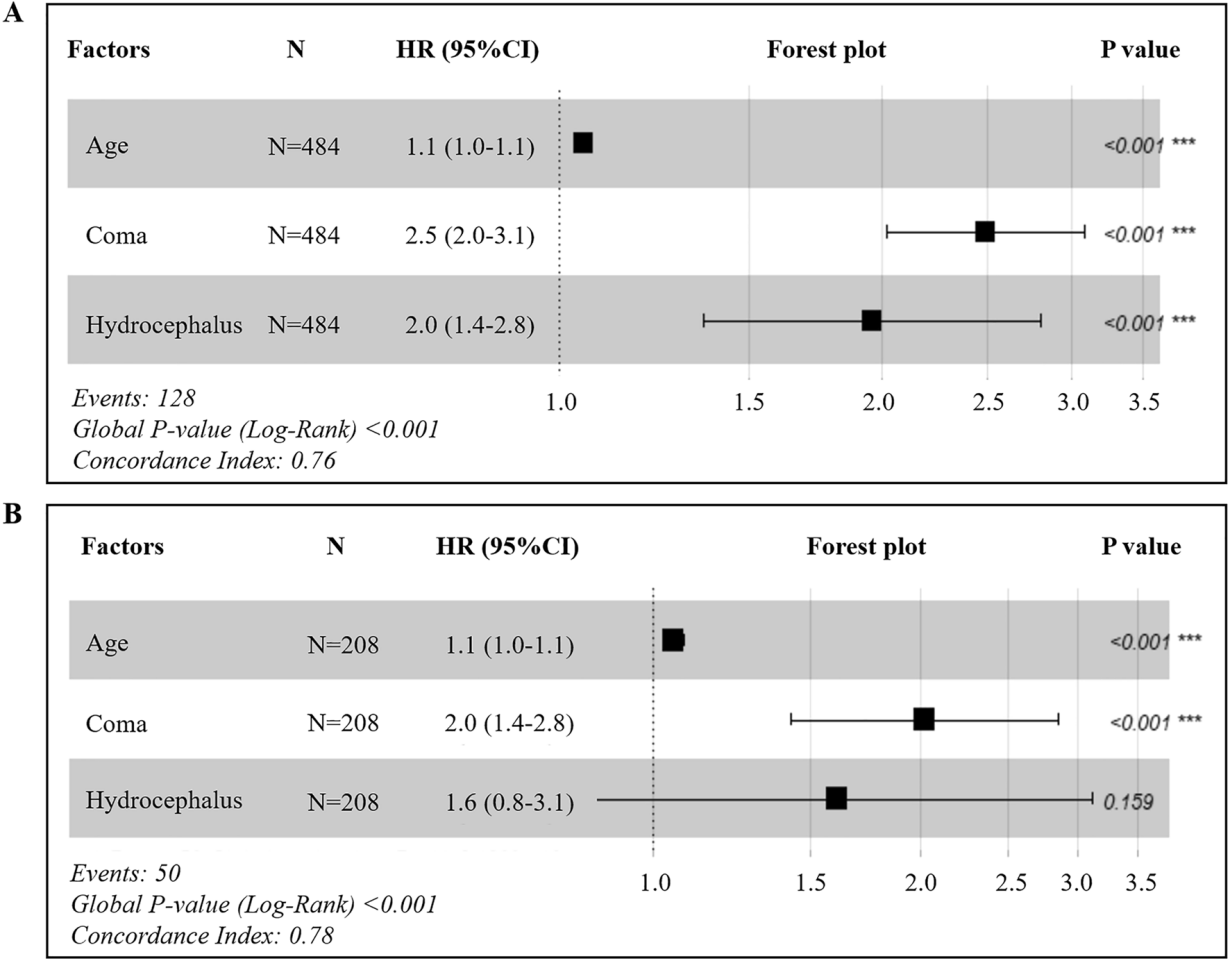
Hazard factors associated with death in sICH patients. (**A**) Forest plot of training set. (**B**) Forest plot of validation set. HR, Hazard Ratio. COX proportional analysis was performed with Survival R package version 3.3–1 (Threneau 2022) and Survminer R package version 0.4.9 (Kassambara et al. 2021). Forest plots were generated with ggplot2 R library (Wickham 2016).

**Figure 4:**
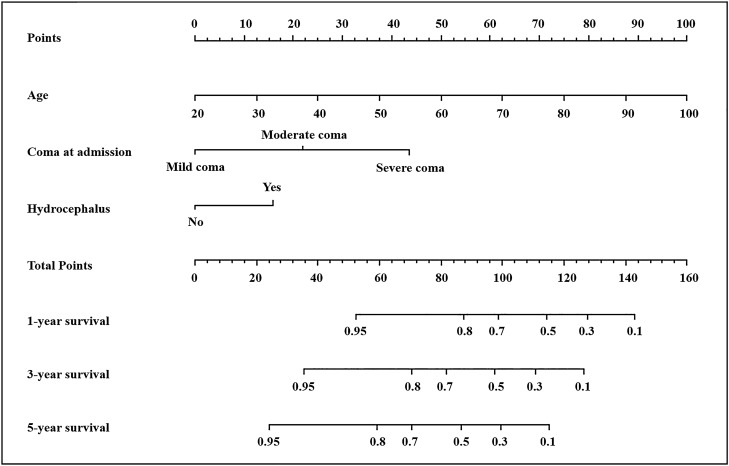
Nomogram for predicting long-term survival in patients with sICH. Survival analysis was performed with Survival R package version 3.3–1 (Threneau 2022). Regression modeling strategies were performed with Rms version 6.3–0 (Harrell 2022).

The results from the univariate analysis and multivariate analysis based on the training cohort were shown in Table 1 and Supplementary Table 3. The COX proportional analysis model (Fig. 3) suggested that increased risk of death in patients with sICH was associated with increased age at onset (HR 1.055, 95% CI 1.038–1.071, *P* < 0.001), the elevated of GCS classification at admission (HR 2.496, 95% CI 2.014–3.093, *P* < 0.001), and the concurrent hydrocephalus (HR 1.955, 95% CI 1.362–2.806, *P* < 0.001). A nomogram constructed from these three risk factors was showed in Fig. 4, which can be used to predict patient survival at 1, 3, and 5 years.

**Table 1:** Univariate and multivariate analysis of death-related factors in patients with cerebral hemorrhage in the training set.

Candidate factors	Survival population (n = 356)	Death population (n = 128)	Model 1^a^		Model 2^b^		Model 3^c^	
			Crude HR (95% CI)	*P*^a^	Adjusted HR1 (95% CI)	*P*^b^	Adjusted HR2 (95% CI)	*P*^c^
Age [years]	58.15 ± 11.65	67.07 ± 11.84	1.049 (1.034–1.064)	< 0.001	1.090 (1.066–1.115)	< 0.001	1.055 (1.038–1.071)	** < 0.001**
GCS								
Mild coma	253 (71.1)	49 (38.3)	2.237 (1.827–2.738)	< 0.001	1.721 (1.161–2.551)	0.007	2.496 (2.014–3.093)	** < 0.001**
Moderate coma	63 (17.7)	33 (25.8)	–	–	–	–	–	–
Severe coma	40 (11.2)	46 (35.9)	–	–	–	–	–	–
Hydrocephalus								
No	296 (83.1)	69 (53.9)	1	–	1	–	1	–
Yes	60 (16.9)	59 (46.1)	3.092 (2.183–4.378)	< 0.001	2.418 (1.449–4.033)	0.001	1.955 (1.362–2.806)	** < 0.001**

^a^Crude HR value and *P* value was calculated by univariate COX proportional hazard model.

^b^HR value and *P* value was calculated by multivariate cox proportional hazard model, adjusted by all meaningful variables in univariate COX proportional hazard model.

^c^HR value and *P* value was calculated by multivariate COX proportional hazard model, adjusted by age, GCS and hydrocephalus.

Significant values are in [bold].

### Validation of the nomogram predictive model

**Figure 5:**
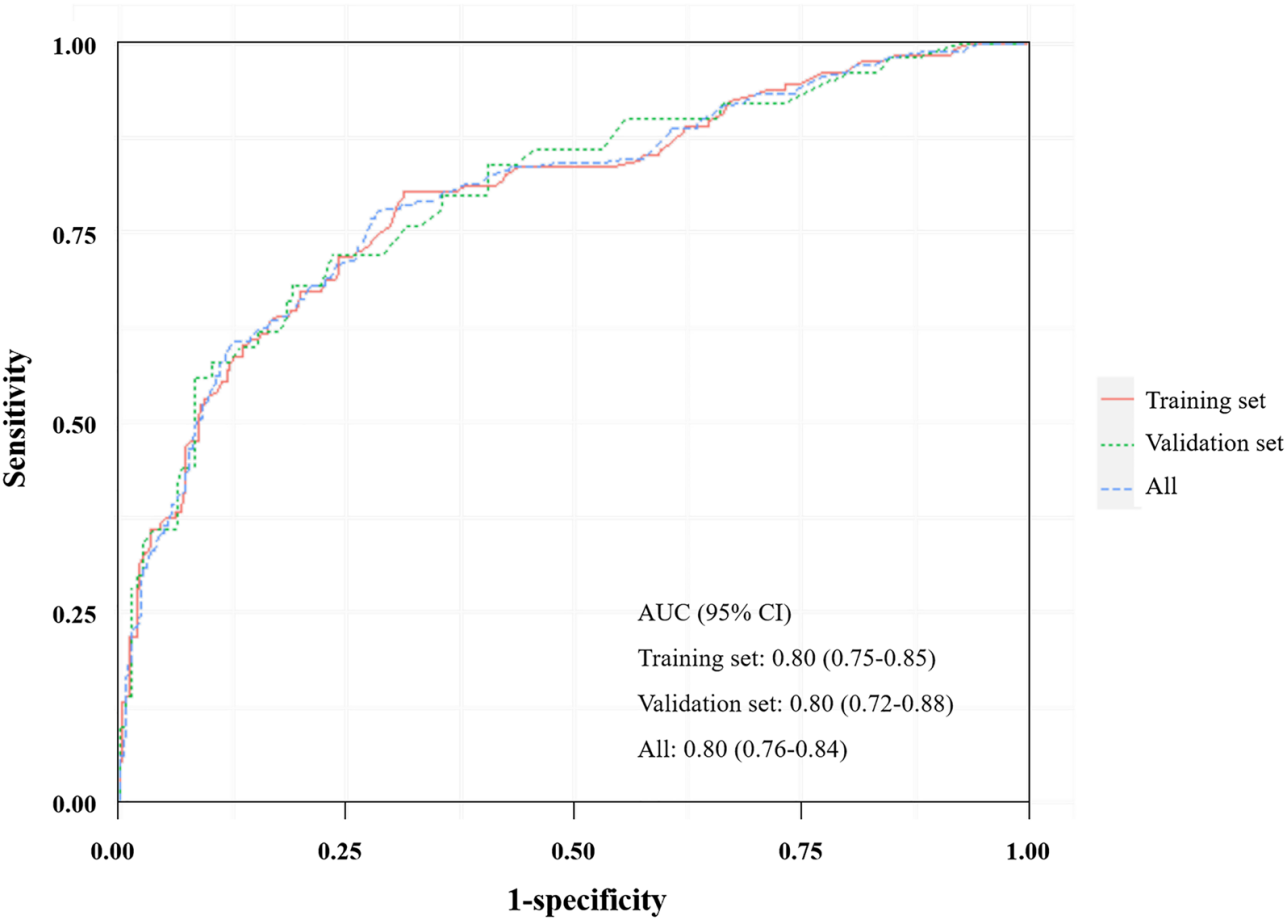
ROC curves for predictive model. The red line represents the ROC curve constructed based on the training set (n = 484); The green line represents the ROC curve constructed based on the validation set (n = 208); The blue line represents the ROC curve constructed based on the all population (n = 692). ROC curves were performed with ROCR (Sing et al. 2005).

The C-Index of predictive model was 0.76 and 0.78 in the training and validation cohorts, respectively. The ROC curves were shown in Fig. 5, and the AUC was 0.80 (95% CI 0.75–0.85) in the training cohort and was 0.80 (95% CI 0.72–0.88) in the validation cohort (Fig. 5). There was no statistical difference between the ROC curves constructed based on the two data sets (*P* > 0.05), indicating that the models were robust. In the training cohort and the validation cohort, the calibration plot for the probability of OS at 1 year, 3 years, or 5 years after discharge showed an optimal agreement between the prediction by nomogram model and actual observation (Supplementary Fig. 1). In addition, the decision curve analysis for the nomogram model in the training cohort and the validation cohort were shown in Supplementary Fig. 2, suggesting that the benefit of the model was better than that of the extreme curve (all patients dead scheme or none patient dead scheme). Moreover, it can be seen that the decision curves constructed by the two data sets have good consistency in 1-year, 3-years, and 5-years.

### Risk score calculation

**Figure 6:**
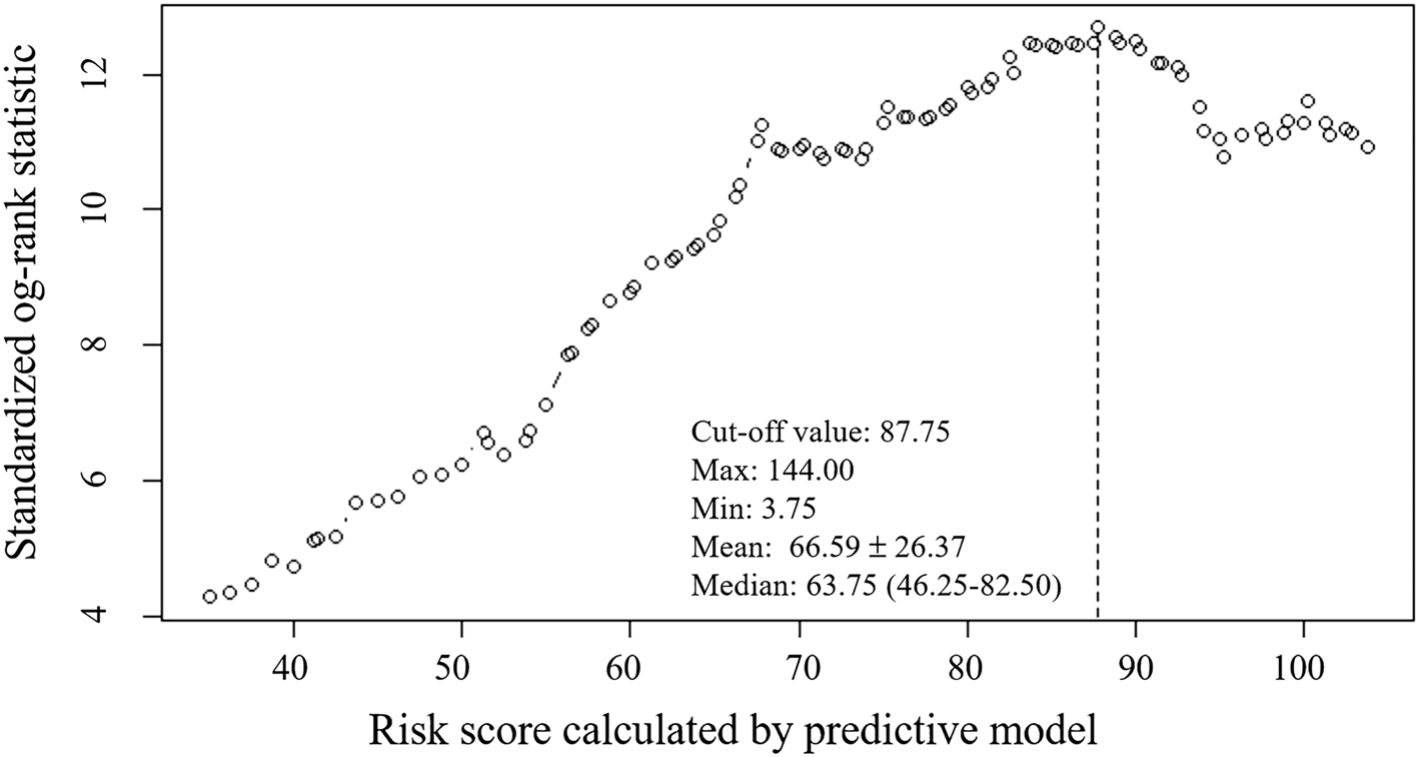
Distribution of risk scores and its best cut-off value. Small circles represent the risk score of each patient. The dotted line represents the location of the cutoff value (cutoff value = 87.75). Graphics were generated with Maxstat R package version 0.7–25 (Hothorn T 2017).

**Figure 7:**
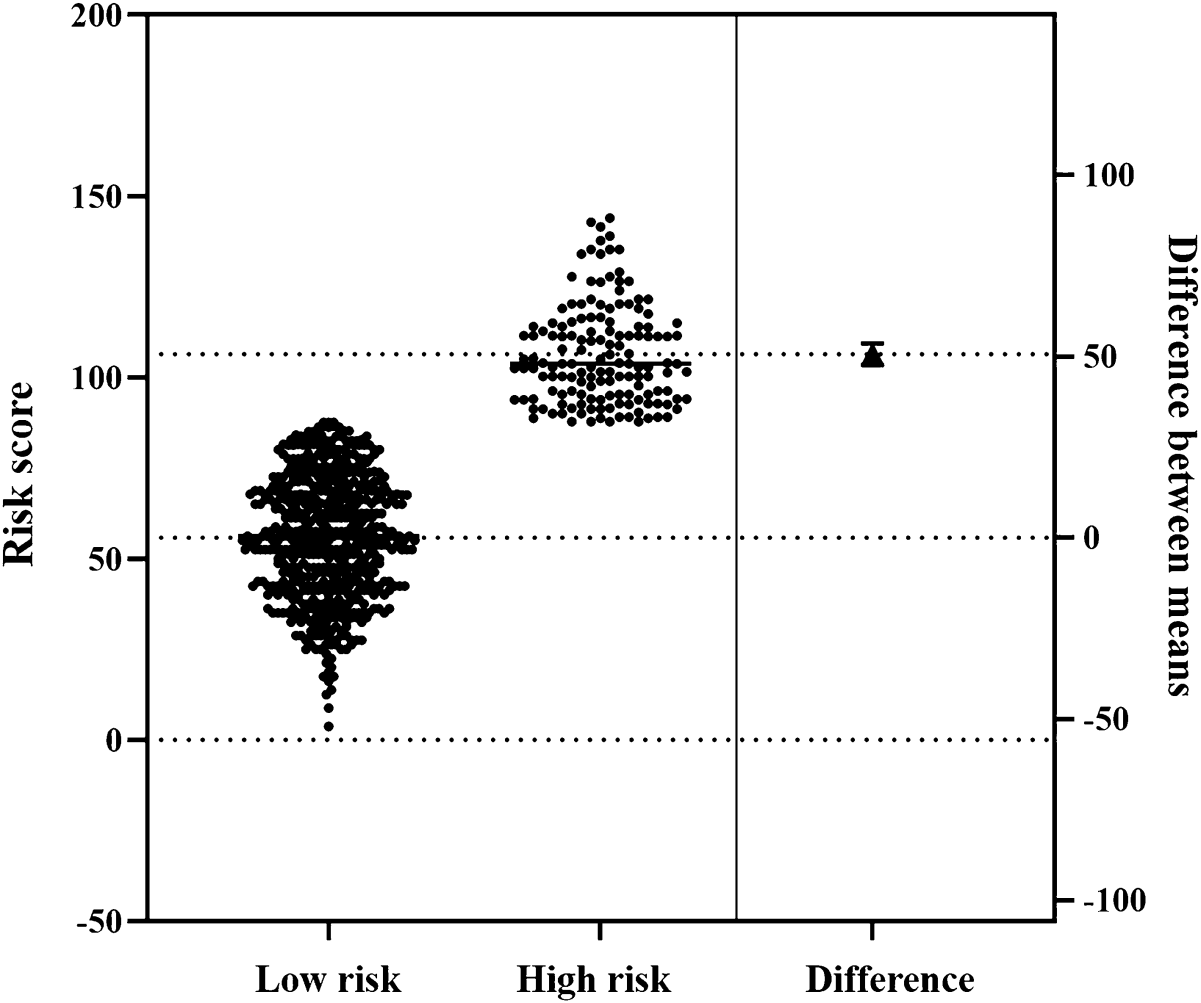
Description of risk scores in low-risk group and high-risk group. The small dots represent the risk score for each patient; The triangle represents the difference of the average scores between the two groups.

The formula for calculating the risk score based on nomogram model was as follows: *Risk score* = (*Age*-20) × 1.25 + *GCS* × 22.5 + *hydrocephalus* × 16.5. Mild coma (GCS 13–15) was assigned a value of 0, and moderate coma (GCS 8–12) was assigned a value of 1, and severe coma (GCS 3–8) was assigned a value of 2. The value is 1 for hydrocephalus patients, and 0 for non-hydrocephalus patients. The value of age ranges from 20 to100. The total risk scores of 692 sICH patients ranged from 3.75 to 144.00. According to the maximally selected rank statistics, subjects were divided into high-risk groups (n = 148) and low-risk groups (n = 544) at a cut-off point of 87.75 (Figs. 6, 7).

### Survive analysis

**Figure 8:**
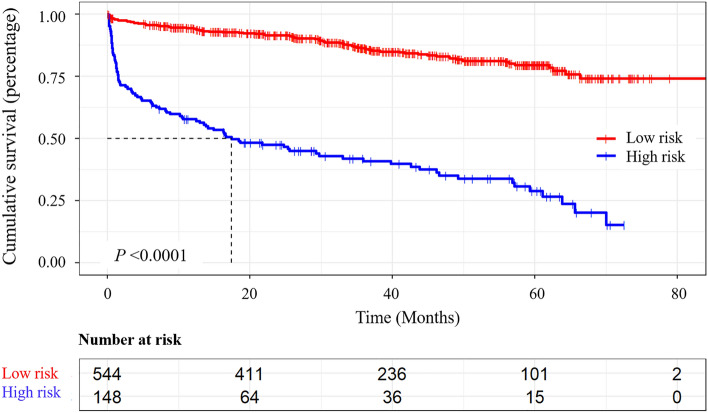
Kaplan–Meier curve of Risk stratification. Red line represents the survival curve of the low-risk group; Blue line represents the survival curve of the high-risk group; The dotted line refers to the median survival time in the high-risk group. Survival analysis was performed with Survival R package version 3.3–1 (Threneau 2022). Survival curves was generated with Survminer R package version 0.4.9 (Kassambara et al. 2021) and ggplot2 R library (Wickham 2016).

In the high-risk group, the mean survival time was 31.01 (26.06–35.95) months and the survival rates of 1 to 6 years were 57.98%, 47.84%, 41.34%, 35.61%, 29.38% and 15.50%, respectively. In the low-risk group, the mean survival time was 72.87 (70.28–75.46) months, and the survival rates of 1 to 6 years were 94.43%, 91.69%, 86.57%, 82.97%, 79.93% and 74.77%, respectively. Log-rank test showed that the survival rate of the high-risk group was significantly lower than that of the low-risk group (Fig. 8, *Log-rank χ*^2^ = 200.03, *P* < 0.001). Compared with the low-risk group, patients in the high-risk group had longer hospital stays (*P* = 0.024) and more expensive hospital charges (*P* < 0.001). In the high-risk group, 47.97% of the patients had a history of ICU treatment (*P* < 0.001), and 22.97% of the patients had undergone an emergency treatment (*P* < 0.001), which was significantly higher than that in the low-risk group (Table 2). The distribution of MRS scores was statistically different between the two groups. The MRS score of the high-risk group [5 (4–5)] was higher than that of the low-risk group [3 (2–4)], and the most patients in high-risk group had a score of 5, accounting for 52.03%.

**Table 2:** Survival information of each risk stratification.

Stratification	Low-risk (N = 544)	High-risk (N = 148)	Statistics	*P* value
Mean survival time	72.87 (70.28–75.46)	31.01 (26.06–35.95)	–	–
1 year survival	94.43%	57.98%	Log Rank *χ*^*2*^ = 200.03	** < 0.001**
2 years survival	91.69%	47.84%		
3 years survival	86.57%	41.34%		
4 years survival	82.97%	35.61%		
5 years survival	79.93%	29.38%		
≥ 6 years survival	74.77%	15.50%		
Days in hospital	16 (12–20.75)	18 (11–27)	− 2.225	**0.024**
Hospital costs (million)	2.35 (1.53–4.25)	5.53 (2.76–9.32)	− 8.305	** < 0.001**
**ICU**				
No	450 (82.72)	77 (52.03)	60.362	** < 0.001**
Yes	94 (17.28)	71 (47.97)	–	**–**
**Emergency**				
No	507 (93.20)	114 (77.03)	33.046	** < 0.001**
Yes	37 (6.80)	34 (22.97)	–	**–**
MRS score at discharge	3 (2–4)	5 (4–5)	− 10.613	** < 0.001**
**MRS Classification at discharge**				
0	9 (1.65)	1 (0.68)	156.612	** < 0.001**
1	95 (17.46)	5 (3.38)		
2	67 (12.32)	7 (4.73)		
3	127 (23.35)	10 (6.76)		
4	192 (35.29)	46 (31.08)		
5	50 (9.19)	77 (52.03)		
6	4 (0.74)	2 (1.35)		

Significant values are in [bold].

## Discussion

### Cohort characteristics and study enlightenment

Many studies have reported considerable variation in the 1-year survival rate between 41 and 70.4% in general ICH patients^22–27^. Only few studies have reported the long-term (more than 3 years) survival rate in ICH patients^22^. The 1-year survival rate in the present study (86.61%) was in the higher range compared with other large sample size studies. This could be attributed to the differences between study subjects and designs. This study only enrolled sICH patients without cerebral herniation at admission, and the majority of them were awake or with mild disturbance of consciousness at admission. On the other hand, the 1-year survival rate in this study was comparable to the 1-year survival rate of MISTIE III (73–81%) and the 6-month survival rate of STICH II (76–82%), which focused on surgical effect on patients without herniation^5,11^. It has become commonplace in both the neurosurgical and neurocritical care literature to presume poor prognosis in ICH patients based on historically quoted mortality rates in general ICH population^26^. However, according to the good long-term survival rate in this study, future studies to determine the effect of surgery on outcomes of sICH patients without acute cerebral herniation may need a more accurate patient selection and grouping, or a larger sample size than MISTIE and STICH research series.

### Admission model

Many ICH prognostic models for mortality and functional outcome have been proposed and validated previously^10–13,22–26,28,29^. However, the vast majority studies only focused on the short-term (within 12 months) mortality, and the long-term survival associated factors were not well defined^8,29^. In the literature, the most frequent predictors for poor outcome were involved in increasing age, decreasing GCS score, increasing hematoma volume, presence of IVH, and deep/infratentorial hematoma location, which were also the principal components of the ICH score^8,23,26^. Increasing age and decreasing GCS score have been the most consistent determinants of long-term outcome, that was in agreement with our results. But, IVH and hematoma volume were not included in the admission model in this study. IVH was univariately related to long-term survival, but was not an independent risk factor in the multivariate model. It was possible that acute hydrocephalus was caused by intraventricular hemorrhage, and that two had an interactive effect on patient prognosis. Once controlling for the interactive effect, IVH no longer remained significant in the predictive model^11^. Hematoma volume was consistently associated with outcome in many reported ICH prediction models, but we did not identify the volume as an independent predictor of long-term survival. This may be attributed to the following two reasons: (1) Previous studies of long-term outcome predictors had mainly considered ICH patients in general, with a wide range of illness severity and potential of survival, that cannot be directly compared with a selected population without acute cerebral herniation; (2) Hematoma volume was strongly correlated with GCS, IVH, and hematoma location. Once adjusted for other variables, the effect of hematoma volume for long-term survival lost its statistical significance. Given that hematoma volume was a significant predictor in multiple prior assessment tools, we re-evaluated our model by entering hematoma volume as a predictive variable. After this modification, hematoma volume showed a modest effect on survival outcome but still lacked significance (OR 0.8; 95% CI 0.4–1.4) in the model development subset. Furthermore, entering hematoma volume into our model did not improve its goodness of fit (The C-index increased from 0.76 to 0.77).

### Clinical relevance

This large sample size retrospective study investigated predictors and verified the nomogram predictive model of long-term mortality (41.77 ± 0.85 months) in supratentorial sICH patients without cerebral herniation at admission. Among the baseline and emergent variables, age, GCS at admission, and hydrocephalus caused by IVH were significantly associated with long-term survival. The admission model, derived from the baseline variables, proved to be a reliable predictor of long-term survival outcome, which was useful in the evaluation and management of patients at the acute phase. The nomogram predictive model was inner verified and shown to be effective and easy to use at the bedside. SICH patients with admission model scores greater than 87.75 were considered to be at high risk of short survival time after hemorrhage and closer monitoring or more effective interventions may be required.

## Conclusions

For sICH patients without cerebral herniation at admission, our de novo nomogram model based on age, GCS and hydrocephalus on CT may be useful to stratify the long-term survival outcomes and provide suggestions for treatment decision-making. SICH patients with admission model scores greater than 87.75 are considered to be at high risk of short survival time, and close observation and active intervention may be needed. The results will be confirmed in our future studies by external validation and prospective cohort.

## Supplementary Information

Supplementary Information.

## Data Availability

The supplementary material for this article can be found online. All processed data and R codes used in this study can be obtained from the corresponding author on reasonable request.
