# Supplementary material for: A nomogram predictive model for long-term survival in spontaneous intracerebral hemorrhage patients without cerebral herniation at admission
Source: Sci Rep. 2023 Feb 22;13:3126. doi: 10.1038/s41598-022-26176-0 (PMC9946945; doi:10.1038/s41598-022-26176-0)
Supplement: Supplementary file 1 — Supplementary Information. [file 41598_2022_26176_MOESM1_ESM.docx]

**Supplementary Table 1.** Basic characteristics of the study population.

| Study factors | Study population  (n=692) | | patients lost to follow-up (n=131) | General population (n=823) | Statistics | *P* |
| --- | --- | --- | --- | --- | --- | --- |
| onset-to-hospital interval [hours] | 12 (6-24) | | 14 (8-24) | 12 (6-24) | -1.899 | 0.058 |
| duration of diabetes [years] | 5 (3-10) | | 6.5 (2.75-10) | 4 (0.2-9) | -0.893 | 0.372 |
| duration of hypertension [years] | 6 (3-10) | | 7 (3-10) | 6 (3-10) | -0.167 | 0.868 |
| systolic pressure [mmHg] | 160 (141-176.75) | | 160 (142-181) | 160 (141.75-177) | -0.690 | 0.490 |
| diastolic pressure [mmHg] | 90 (80-102) | | 91 (80-102.5) | 90 (80-102) | -0.457 | 0.648 |
| blood urea nitrogen [mmol/L] | 4.9 (4.04-6) | | 4.71 (3.83-6.22) | 4.88 (3.98-6) | -0.881 | 0.378 |
| uric acid [μmol/L] | 321.4 (258.5-405.15) | | 323 (257.5-394) | 321.5 (258.75-402.78) | -0.205 | 0.838 |
| hematoma volume [ml] | 12.83 (5.715-27.948) | | 12.73 (5.7-22.62) | 12.76 (5.7-27.6) | -0.894 | 0.371 |
| edema volume [ml] | 10.55 (4.57-20.80) | | 9.83 (4.54-19.89) | 10.4 (4.57-20.66) | -0.633 | 0.527 |
| age | 78.26 ± 15.08 | | 77.15 ± 13.15 | 78.08 ± 14.79 | 0.787 | 0.431 |
| temperature [℃] | 60.45 ± 12.32 | | 57.35 ± 11.62 | 59.95 ± 12.26 | 2.768 | 0.006 |
| heart rate [times / minute] | 36.66 ± 0.40 | | 36.6 ± 0.31 | 36.65 ± 0.39 | 1.484 | 0.138 |
| breath rate [times / minute] | 20.25 ± 6.31 | | 20.26 ± 6.14 | 20.26 ± 6.28 | -0.012 | 0.990 |
| white blood cells [10^9/L] | 9.65 ± 3.71 | | 9.43 ± 3.27 | 9.61 ± 3.64 | 0.591 | 0.555 |
| neutrophils [10^9/L] | 7.98 ± 6.11 | | 7.54 ± 3.31 | 7.91 ± 5.75 | 0.776 | 0.438 |
| lymphocytes [10^9/L] | 1.33 ± 1.22 | | 1.36 ± 1.42 | 1.34 ± 1.25 | -0.275 | 0.783 |
| red blood cells [10^12/L] | 4.60 ± 0.59 | | 4.57 ± 0.55 | 4.59 ± 0.59 | 0.513 | 0.608 |
| hemoglobin [g/L] | 140.51 ± 17.62 | | 146.98 ± 109.16 | 141.53 ± 46.29 | -0.648 | 0.518 |
| hematocrit [L/L] | 0.95 ± 9.92 | | 0.40 ± 0.05 | 0.87 ± 9.10 | 0.607 | 0.544 |
| platelets [10^9/L] | 209.61 ± 68.73 | | 217.82 ± 70.80 | 210.91 ± 69.08 | -1.194 | 0.233 |
| blood glucose [mmol/L] | 7.18 ± 3.15 | | 7.25 ± 3.10 | 7.19 ± 3.14 | -0.237 | 0.813 |
| K+ [mmol/L] | 3.83 ± 0.43 | | 3.78 ± 0.51 | 3.82 ± 0.45 | 1.005 | 0.316 |
| Na+ [mmol/L] | 140.17 ± 4.00 | | 140.03 ± 4.27 | 140.15 ± 4.04 | 0.364 | 0.716 |
| Cl- [mmol/L] | 100.75 ± 16.78 | | 100.89 ± 16.56 | 100.78 ± 16.73 | -0.085 | 0.932 |
| anion gap [mmol/L] | 12.54 ± 5.14 | | 12.18 ± 3.71 | 12.48 ± 4.94 | 0.738 | 0.460 |
| prothrombin time [seconds] | 11.60 ± 2.15 | | 11.59 ± 2.40 | 11.59 ± 2.19 | 0.040 | 0.968 |
| PT-INR | 0.97 ± 0.28 | | 0.95 ± 0.32 | 0.97 ± 0.29 | 0.586 | 0.558 |
| fibrinogen [g/L] | 2.76 ± 0.83 | | 2.77 ± 0.86 | 2.76 ± 0.83 | -0.158 | 0.875 |
| thrombin time [seconds] | 17.90 ± 9.27 | | 17.39 ± 1.55 | 17.82 ± 8.51 | 0.596 | 0.552 |
| sex | Female | 204 (29.5) | 39 (29.8) | 243 (29.5) | 0.004 | 1.000 |
|  | Male | 488 (70.5) | 92 (70.2) | 580 (70.5) |  |  |
| consciousness disorder | No | 380 (54.9) | 80 (61.1) | 460 (55.9) | 1.693 | 0.213 |
|  | Yes | 312 (45.1) | 51 (38.9) | 363 (44.1) |  |  |
| epilepsy | No | 669 (96.7) | 129 (98.5) | 798 (97.0) | 1.208 | 0.406 |
|  | Yes | 23 (3.3) | 2 (1.5) | 25 (3.0) |  |  |
| headache | No | 462 (66.8) | 92 (70.2) | 554 (67.3) | 0.601 | 0.478 |
|  | Yes | 230 (33.2) | 39 (29.8) | 269 (32.7) |  |  |
| neurological dysfunction | No | 122 (17.6) | 26 (19.8) | 148 (18.0) | 0.367 | 0.620 |
|  | Yes | 570 (82.4) | 105 (80.2) | 675 (82.0) |  |  |
| smoke | No | 520 (75.3) | 103 (78.6) | 623 (75.8) | 0.683 | 0.438 |
|  | Yes | 171 (24.7) | 28 (21.4) | 199 (24.2) |  |  |
| drink | No | 525 (75.9) | 102 (77.9) | 627 (76.2) | 0.242 | 0.656 |
|  | Yes | 167 (24.1) | 29 (22.1) | 196 (23.8) |  |  |
| medical history |  |  |  |  |  |  |
| diabetes | No | 615 (88.9) | 111 (84.7) | 726 (88.2) | 1.816 | 0.184 |
|  | Yes | 77 (11.1) | 20 (15.3) | 97 (11.8) |  |  |
| control of diabetes | No | 615 (88.9) | 111 (84.7) | 726 (88.2) |  | 0.330 |
|  | Good | 8 (1.2) | 3 (2.3) | 11 (1.3) |  |  |
|  | Poor | 8 (1.2) | 1 (0.8) | 9 (1.1) |  |  |
|  | Uncontrolled | 5 (0.7) | 0 | 5 (0.6) |  |  |
|  | Unknown | 56 (8.1) | 16 (12.2) | 72 (8.7) |  |  |
| hypertension | No | 89 (12.9) | 16 (12.2) | 105 (12.8) | 0.042 | 0.988 |
|  | Past diagnosed | 498 (72.0) | 95 (72.5) | 593 (72.1) |  |  |
|  | Newly diagnosed | 105 (15.2) | 20 (15.3) | 125 (15.2) |  |  |
| control of hypertension | No | 194 (28.0) | 36 (27.5) | 230 (27.9) |  | 0.035 |
|  | Good | 28 (4.0) | 3 (2.3) | 31 (3.8) |  |  |
|  | Poor | 54 (7.8) | 2 (1.5) | 56 (6.8) |  |  |
|  | Uncontrolled | 46 (6.6) | 6 (4.6) | 52 (6.3) |  |  |
|  | Unknown | 370 (53.5) | 84 (64.1) | 454 (55.2) |  |  |
| dyslipidemia | No | 687 (99.3) | 130 (99.2) | 817 (99.3) | 0.000 | 1.000 |
|  | Yes | 5 (0.7) | 1 (0.8) | 6 (0.7) |  |  |
| heart diseases | No | 663 (95.8) | 123 (93.9) | 786 (95.5) | 0.942 | 0.355 |
|  | Yes | 29 (4.2) | 8 (6.1) | 37 (4.5) |  |  |
| hematological diseases | No | 690 (99.7) | 131 (100) | 821 (99.8) |  | 1.000 |
|  | Yes | 2 (0.3) | 0 | 2 (0.2) |  |  |
| cerebrovascular diseases | No | 642 (92.8) | 123 (93.9) | 765 (93.0) | 0.210 | 0.715 |
|  | Yes | 50 (7.2) | 8 (6.1) | 58 (7.0) |  |  |
| dementia | No | 686 (99.3) | 131 (100) | 817 (99.4) |  | 1.000 |
|  | Yes | 5 (0.7) | 0 | 5 (0.6) |  |  |
| liver diseases | No | 665 (96.1) | 128 (97.7) | 793 (96.4) |  | 0.456 |
|  | Yes | 27 (3.9) | 3 (2.3) | 30 (3.6) |  |  |
| premobid mRS | 0 | 602 (87.0) | 119 (90.8) | 721 (87.6) | 1.500 | 0.250 |
|  | 1 | 90 (13.0) | 12 (9.2) | 102 (12.4) |  |  |
| family medical history |  |  |  |  |  |  |
| hypertension | No | 647 (93.8) | 122 (93.8) | 769 (93.8) | 0.001 | 1.000 |
|  | Yes | 43 (6.2) | 8 (6.2) | 51 (6.2) |  |  |
| hyperlipidemia | No | 690 (100) | 130 (100) | 820 (100) | -- | -- |
|  | Yes | 0 | 0 | 0 |  |  |
| diabetes | No | 682 (98.8) | 127 (97.7) | 809 (98.7) | 0.395 | 0.53a |
|  | Yes | 8 (1.2) | 3 (2.3) | 11 (1.3) |  |  |
| stroke | No | 670 (97.1) | 127 (97.7) | 797 (97.2) | 0.007 | 0.932a |
|  | Yes | 20 (2.9) | 3 (2.3) | 23 (2.8) |  |  |
| coronary heart disease | No | 689 (99.9) | 129 (99.2) | 818 (99.8) |  | 0.292 |
|  | Yes | 1 (0.1) | 1 (0.8) | 2 (0.2) |  |  |
| Medication history |  |  |  |  |  |  |
| antiplatelet drugs | No | 677 (98.0) | 126 (96.2) | 803 (97.7) | 0.871 | 0.351 |
|  | Yes | 14 (2.0) | 5 (3.8) | 19 (2.3) |  |  |
| anticoagulant drugs | No | 684 (98.8) | 130 (99.2) | 814 (98.9) | 0.000 | 1.000 |
|  | Yes | 8 (1.2) | 1 (0.8) | 9 (1.1) |  |  |
| antihypertensive drugs | No | 300 (44.8) | 58 (45.7) | 358 (45.0) | 0.029 | 0.923 |
|  | Yes | 369 (55.2) | 69 (54.3) | 438 (55.0) |  |  |
| left pupil shape | Circular | 688 (99.6) | 130 (99.2) | 818 (99.5) |  | 0.501 |
|  | Non-circular | 3 (0.4) | 1 (0.8) | 4 (0.5) |  |  |
| right pupil shape | Circular | 690 (100) | 131 (100) | 821 (100) | -- | -- |
|  | Non-circular | 0 | 0 | 0 |  |  |
| GCS classification | Mild coma | 436 (63.0) | 88 (67.2) | 524 (63.7) | 1.487 | 0.479 |
|  | Moderate coma | 131 (18.9) | 25 (19.1) | 156 (19.0) |  |  |
|  | Severe coma | 125 (18.1) | 18 (13.7) | 143 (17.4) |  |  |
| mRS | 0 | 1 (0.1) | 0 | 1 (0.1) | 3.671 | 0.565 |
|  | 1 | 32 (4.6) | 4 (3.1) | 36 (4.4) |  |  |
|  | 2 | 56 (8.1) | 13 (9.9) | 69 (8.4) |  |  |
|  | 3 | 98 (14.2) | 12 (9.2) | 110 (13.4) |  |  |
|  | 4 | 286 (41.3) | 58 (44.3) | 344 (41.8) |  |  |
|  | 5 | 219 (31.6) | 44 (33.6) | 263 (32.0) |  |  |
| subarachnoid hemorrhage | No | 646 (93.4) | 125 (95.4) | 771 (93.7) | 0.795 | 0.439 |
|  | Yes | 46 (6.6) | 6 (4.6) | 52 (6.3) |  |  |
| position of subarachnoid hemorrhage | | | | | |  |
| supratentorial lobes | No | 508 (73.4) | 105 (80.2) | 613 (74.5) | 2.635 | 0.126 |
|  | Yes | 184 (26.6) | 26 (19.8) | 210 (25.5) |  |  |
| frontal lobe | No | 604 (87.3) | 121 (92.4) | 725 (88.1) | 2.713 | 0.107 |
|  | Yes | 88 (12.7) | 10 (7.6) | 98 (11.9) |  |  |
| temporal lobe | No | 600 (86.7) | 114 (87.0) | 714 (86.8) | 0.010 | 1.000 |
|  | Yes | 92 (13.3) | 17 (13.0) | 109 (13.2) |  |  |
| parietal lobe | No | 602 (87.0) | 123 (93.9) | 725 (88.1) | 4.998 | 0.027 |
|  | Yes | 90 (13.0) | 8 (6.1) | 98 (11.9) |  |  |
| occipital lobe | No | 659 (95.2) | 127 (96.9) | 786 (95.5) | 0.755 | 0.494 |
|  | Yes | 33 (4.8) | 4 (3.1) | 37 (4.5) |  |  |
| hemispheres | No | 508 (73.4) | 105 (80.2) | 613 (74.5) |  | 0.499 |
|  | Left | 83 (12.0) | 12 (9.2) | 95 (11.5) |  |  |
|  | Right | 99 (14.3) | 14 (10.7) | 113 (13.7) |  |  |
|  | Both | 2 (0.3) | 0 | 2 (0.2) |  |  |
| deep supratentorial hemorrhage | No | 120 (17.3) | 12 (9.2) | 132 (16.0) | 5.474 | 0.019 |
|  | Yes | 572 (82.7) | 119 (90.8) | 691 (84.0) |  |  |
| position of deep supratentorial hemorrhage | | | |  |  |  |
| corona radiata | No | 671 (97.0) | 129 (98.5) | 800 (97.2) | 0.922 | 0.408 |
|  | Yes | 21 (3.0) | 2 (1.5) | 23 (2.8) |  |  |
| basal ganglia | No | 220 (31.8) | 28 (21.4) | 248 (30.1) | 5.678 | 0.022 |
|  | Yes | 472 (68.2) | 103 (78.6) | 575 (69.9) |  |  |
| thalamus | No | 507 (73.3) | 96 (73.3) | 603 (73.3) | 0.000 | 1.000 |
|  | Yes | 185 (26.7) | 35 (26.7) | 220 (26.7) |  |  |
| insula | No | 672 (97.1) | 130 (99.2) | 802 (97.4) | 1.240 | 0.266 |
|  | Yes | 20 (2.9) | 1 (0.8) | 21 (2.6) |  |  |
| hemispheres | No | 120 (17.3) | 12 (9.2) | 132 (16.0) |  | 0.085 |
|  | Left | 292 (42.2) | 57 (43.5) | 349 (42.4) |  |  |
|  | Right | 278 (40.2) | 62 (47.3) | 340 (41.3) |  |  |
|  | Both | 2 (0.3) | 0 | 2 (0.2) |  |  |
| intraventricular hemorrhage† | No | 441 (63.7) | 88 (67.2) | 529 (64.3) | 0.570 | 0.487 |
|  | Yes | 251 (36.3) | 43 (32.8) | 294 (35.7) |  |  |
| position of intraventricular hemorrhage | | | | | | |
| lateral ventricular hemorrhage† | No | 445 (64.3) | 88 (67.2) | 533 (64.8) | 0.397 | 0.551 |
|  | Yes | 247 (35.7) | 43 (32.8) | 290 (35.2) |  |  |
| hemispheres | No | 445 (64.3) | 88 (67.2) | 533 (64.8) | 1.028 | 0.797 |
|  | Left | 50 (7.2) | 10 (7.6) | 60 (7.3) |  |  |
|  | Right | 43 (6.2) | 9 (6.9) | 52 (6.3) |  |  |
|  | Both | 154 (22.3) | 24 (18.3) | 178 (21.6) |  |  |
| third ventricle | No | 575 (83.1) | 112 (85.5) | 687 (83.5) | 0.461 | 0.525 |
|  | Yes | 117 (16.9) | 19 (14.5) | 136 (16.5) |  |  |
| fourth ventricle | No | 591 (85.4) | 121 (92.4) | 712 (86.5) | 4.575 | 0.036 |
|  | Yes | 101 (14.6) | 10 (7.6) | 111 (13.5) |  |  |
| compression of periventricular tissues | No | 166 (24.0) | 25 (19.1) | 191 (23.2) | 1.487 | 0.259 |
|  | Yes | 526 (76.0) | 106 (80.9) | 632 (76.8) |  |  |
| hydrocephalus | No | 539 (77.9) | 110 (84.0) | 649 (78.9) | 2.442 | 0.130 |
|  | Yes | 153 (22.1) | 21 (16.0) | 174 (21.1) |  |  |
| CT/CTA signs |  |  |  |  |  |  |
| degree of margin irregularity | 1 | 287 (41.6) | 54 (41.2) | 341 (41.5) | 0.301 | 0.991 |
|  | 2 | 206 (29.9) | 38 (29.0) | 244 (29.7) |  |  |
|  | 3 | 88 (12.8) | 17 (13.0) | 105 (12.8) |  |  |
|  | 4 | 40 (5.8) | 7 (5.3) | 47 (5.7) |  |  |
|  | 5 | 69 (10.0) | 15 (11.5) | 84 (10.2) |  |  |
| degree of density heterogeneity | 1 | 416 (60.3) | 79 (60.3) | 495 (60.3) | 2.450 | 0.653 |
|  | 2 | 193 (28.0) | 42 (32.1) | 235 (28.6) |  |  |
|  | 3 | 54 (7.8) | 6 (4.6) | 60 (7.3) |  |  |
|  | 4 | 13 (1.9) | 2 (1.5) | 15 (1.8) |  |  |
|  | 5 | 14 (2.0) | 2 (1.5) | 16 (1.9) |  |  |
| black hole sign | No | 641 (92.6) | 125 (95.4) | 766 (93.1) | 1.330 | 0.270 |
|  | Yes | 51 (7.4) | 6 (4.6) | 57 (6.9) |  |  |
| island sign | No | 641 (92.6) | 122 (93.1) | 763 (92.7) | 0.041 | 0.859 |
|  | Yes | 51 (7.4) | 9 (6.9) | 60 (7.3) |  |  |
| swirl sign | No | 668 (96.5) | 127 (96.9) | 795 (96.6) | 0.000 | 1.000a |
|  | Yes | 24 (3.5) | 4 (3.1) | 28 (3.4) |  |  |
| fluid-blood level within the hematoma | No | 684 (98.8) | 127 (96.9) | 811 (98.5) | 2.760 | 0.108 |
|  | Yes | 8 (1.2) | 4 (3.1) | 12 (1.5) |  |  |
| else | No | 683 (98.7) | 131 (100) | 814 (98.9) | 0.730 | 0.393a |
|  | Yes | 9 (1.3) | 0 | 9 (1.1) |  |  |
| vasculopathy | No | 663 (95.8) | 127 (96.9) | 790 (96.0) | 0.370 | 0.637 |
|  | Yes | 29 (4.2) | 4 (3.1) | 33 (4.0) |  |  |

a *P* value was calculated by the continuity-corrected chi-square test.

**Supplementary Table 2.** Basic characteristics between the train set and validation set.

| study factors |  | Training set (n=484) | Validation set (n=208) | All set (n=692) | *Statistics* | *P* |
| --- | --- | --- | --- | --- | --- | --- |
| onset-to-hospital interval [hours] |  | 12 (7-24) | 10 (6-24) | 12 (6-24) | -2.587 | 0.010 |
| duration of diabetes [years] |  | 5 (3-10) | 5 (1.5-10) | 5 (3-10) | -0.673 | 0.501 |
| duration of hypertension [years] |  | 6 (3-10) | 5 (3-10) | 6 (3-10) | -0.696 | 0.487 |
| systolic pressure [mmHg] | | 158.50 ± 28.97 | 158.71 ± 23.51 | 158.57 ± 27.42 | -0.100 | 0.920 |
| diastolic pressure [mmHg] | | 92.00 ± 15.52 | 90.74 ± 15.41 | 91.62 ± 15.48 | 0.975 | 0.330 |
| serum creatinine [μmol/L] |  | 65.5 (53-77.6) | 68.1 (56.8-81.45) | 66 (54.625-79) | -2.298 | 0.022 |
| blood urea nitrogen [mmol/L] |  | 4.8 (3.84-5.84) | 5.1 (4.305-6.3) | 4.9 (4.04-6) | -3.094 | 0.002 |
| uric acid [μmol/L] |  | 319.9 (254.85-414.2) | 324 (272-392.5) | 321.4 (258.5-405.15) | -0.488 | 0.626 |
| hematoma volume [ml] |  | 12.33 (5.78-27.79) | 13.40 (5.19-28.26) | 12.83 (5.72-27.95) | -0.385 | 0.700 |
| edema volume [ml] |  | 11.07 (4.58-20.94) | 10.23 (4.35-19.89) | 10.55 (4.57-20.80) | -0.716 | 0.474 |
| age [years] |  | 60.24 ± 12.63 | 60.91 ± 11.59 | 60.45 ± 12.32 | -0.655 | 0.513 |
| temperature [℃] |  | 36.66 ± 0.42 | 36.64 ± 0.34 | 36.66 ± 0.40 | 0.641 | 0.522 |
| heart rate [times / minute] | | 78.34 ± 14.91 | 78.06 ± 15.53 | 78.26 ± 15.08 | 0.224 | 0.823 |
| breath rate [times / minute] | | 20.27 ± 6.52 | 20.22 ± 5.81 | 20.25 ± 6.31 | 0.091 | 0.928 |
| white blood cells [10^9/L] |  | 9.57 ± 3.78 | 9.83 ± 3.53 | 9.65 ± 3.71 | -0.814 | 0.416 |
| neutrophils [10^9/L] |  | 7.81 ± 5.37 | 8.37 ± 7.54 | 7.98 ± 6.11 | -1.063 | 0.288 |
| lymphocytes [10^9/L] |  | 1.36 ± 1.37 | 1.26 ± 0.79 | 1.33 ± 1.22 | 0.957 | 0.339 |
| red blood cells [10^12/L] |  | 4.60 ± 0.63 | 4.59 ± 0.51 | 4.60 ± 0.59 | 0.036 | 0.972 |
| hemoglobin [g/L] |  | 140.54 ± 18.20 | 140.42 ± 16.25 | 140.51 ± 17.62 | 0.082 | 0.935 |
| hematocrit [L/L] |  | 0.72 ± 6.52 | 1.50 ± 15.09 | 0.95 ± 9.92 | -0.687 | 0.493 |
| platelets [10^9/L] |  | 207.25 ± 65.29 | 215.03 ± 75.97 | 209.61 ± 68.73 | -1.314 | 0.189 |
| Fasting blood glucose [mol/L] |  | 7.12 ± 3.07 | 7.32 ± 3.34 | 7.18 ± 3.15 | -0.757 | 0.449 |
| K+ [mmol/L] |  | 3.84 ± 0.43 | 3.81 ± 0.44 | 3.83 ± 0.43 | 0.609 | 0.543 |
| Na+ [mmol/L] |  | 140.26 ± 4.00 | 139.95 ± 4.00 | 140.17 ± 4.00 | 0.907 | 0.365 |
| Cl- [mmol/L] |  | 101.80 ± 13.67 | 98.34 ± 22.24 | 100.75 ± 16.78 | 1.991 | 0.048 |
| anion gap [mmol/L] |  | 12.59 ± 5.57 | 12.43 ± 3.99 | 12.54 ± 5.14 | 0.356 | 0.722 |
| prothrombin time [seconds] |  | 11.67 ± 2.46 | 11.44 ± 1.12 | 11.60 ± 2.15 | 1.226 | 0.221 |
| PT-INR | | 0.98 ± 0.28 | 0.94 ± 0.26 | 0.97 ± 0.28 | 1.927 | 0.054 |
| fibrinogen [g/L] |  | 2.73 ± 0.80 | 2.83 ± 0.89 | 2.76 ± 0.83 | -1.484 | 0.138 |
| thrombin time [seconds] |  | 18.06 ± 10.82 | 17.52 ± 3.88 | 17.90 ± 9.27 | 0.678 | 0.498 |
| sex | Female | 149 (30.8) | 55 (26.4) | 204 (29.5) | 1.301 | 0.275 |
|  | Male | 335 (69.2) | 153 (73.6) | 488 (70.5) |  |  |
| consciousness disorder | No | 269 (55.6) | 111 (53.4) | 380 (54.9) | 0.288 | 0.618 |
|  | Yes | 215 (44.4) | 97 (46.6) | 312 (45.1) |  |  |
| epilepsy | No | 467 (96.5) | 202 (97.1) | 669 (96.7) | 0.178 | 0.819 |
|  | Yes | 17 (3.5) | 6 (2.9) | 23 (3.3) |  |  |
| headache | No | 324 (66.9) | 138 (66.3) | 462 (66.8) | 0.023 | 0.930 |
|  | Yes | 160 (33.1) | 70 (33.7) | 230 (33.2) |  |  |
| neurological dysfunction | No | 90 (18.6) | 32 (15.4) | 122 (17.6) | 1.033 | 0.329 |
|  | Yes | 394 (81.4) | 176 (84.6) | 570 (82.4) |  |  |
| smoke | No | 356 (73.6) | 164 (79.2) | 520 (75.3) | 2.506 | 0.124 |
|  | Yes | 128 (26.4) | 43 (20.8) | 171 (24.7) |  |  |
| drink | No | 362 (74.8) | 163 (78.4) | 525 (75.9) | 1.014 | 0.334 |
|  | Yes | 122 (25.2) | 45 (21.6) | 167 (24.1) |  |  |
| medical history |  |  |  |  |  |  |
| diabetes | No | 429 (88.6) | 186 (89.4) | 615 (88.9) | 0.091 | 0.794 |
|  | Yes | 55 (11.4) | 22 (10.6) | 77 (11.1) |  |  |
| control of diabetes | No | 429 (88.6) | 186 (89.4) | 615 (88.9) | 2.996 | 0.579 |
|  | Good | 4 (0.8) | 4 (1.9) | 8 (1.2) |  |  |
|  | Poor | 7 (1.4) | 1 (0.5) | 8 (1.2) |  |  |
|  | Uncontrolled | 4 (0.8) | 1 (0.5) | 5 (0.7) |  |  |
|  | Unknown | 40 (8.3) | 16 (7.7) | 56 (8.1) |  |  |
| hypertension | No | 56 (11.6) | 33 (15.9) | 89 (12.9) | 2.514 | 0.283 |
|  | Past diagnosed | 352 (72.7) | 146 (70.2) | 498 (72.0) |  |  |
|  | Newly diagnosed | 76 (15.7) | 29 (13.9) | 105 (15.2) |  |  |
| control of hypertension | No | 132 (27.3) | 62 (29.8) | 194 (28.0) | 3.542 | 0.474 |
|  | Good | 20 (4.1) | 8 (3.8) | 28 (4.0) |  |  |
|  | Poor | 33 (6.8) | 21 (10.1) | 54 (7.8) |  |  |
|  | Uncontrolled | 35 (7.2) | 11 (5.3) | 46 (6.6) |  |  |
|  | Unknown | 264 (54.5) | 106 (51.0) | 370 (53.5) |  |  |
| dyslipidemia | No | 481 (99.4) | 206 (99.0) | 687 (99.3) |  | 0.640 |
|  | Yes | 3 (0.6) | 2 (1.0) | 5 (0.7) |  |  |
| heart diseases | No | 461 (95.2) | 202 (97.1) | 663 (95.8) | 1.264 | 0.307 |
|  | Yes | 23 (4.8) | 6 (2.9) | 29 (4.2) |  |  |
| hematological diseases | No | 482 (99.6) | 208 (100) | 690 (99.7) | 0.862 | 0.579 |
|  | Yes | 2 (0.4) | 0 | 2 (0.3) |  |  |
| cerebrovascular diseases | No | 451 (93.2) | 191 (91.8) | 642 (92.8) | 0.398 | 0.631 |
|  | Yes | 33 (6.8) | 17 (8.2) | 50 (7.2) |  |  |
| dementia | No | 481 (99.6) | 205 (98.6) | 686 (99.3) |  | 0.164 |
|  | Yes | 2 (0.4) | 3 (1.4) | 5 (0.7) |  |  |
| liver diseases | No | 460 (95.0) | 205 (98.6) | 665 (96.1) | 4.798 | 0.031 |
|  | Yes | 24 (5.0) | 3 (1.4) | 27 (3.9) |  |  |
| premobid mRS | 0 | 425 (87.8) | 177 (85.1) | 602 (87.0) | 0.947 | 0.388 |
|  | 1 | 59 (12.2) | 31 (14.9) | 90 (13.0) |  |  |
| Family history |  |  |  |  |  |  |
| hypertension | No | 448 (92.8) | 199 (96.1) | 647 (93.8) | 2.836 | 0.121 |
|  | Yes | 35 (7.2) | 8 (3.9) | 43 (6.2) |  |  |
| hyperlipidemia | No | 483 (100) | 207 (100) | 690 (100) |  |  |
|  | Yes | 0 | 0 | 0 |  |  |
| diabetes | No | 476 (98.6) | 206 (99.5) | 682 (98.8) | 1.180 | 0.447 |
|  | Yes | 7 (1.4) | 1 (0.5) | 8 (1.2) |  |  |
| stroke | No | 472 (97.7) | 198 (95.7) | 670 (97.1) | 2.207 | 0.214 |
|  | Yes | 11 (2.3) | 9 (4.3) | 20 (2.9) |  |  |
| coronary heart disease | No | 483 (100) | 206 (99.5) | 689 (99.9) |  | 0.300 |
|  | Yes | 0 | 1 (0.5) | 1 (0.1) |  |  |
| Medication history |  |  |  |  |  |  |
| antiplatelet drugs | No | 472 (97.7) | 205 (98.6) | 677 (98.0) | 0.177 | 0.674a |
|  | Yes | 11 (2.3) | 3 (1.4) | 14 (2.0) |  |  |
| anticoagulant drugs | No | 478 (98.8) | 206 (99.0) | 684 (98.8) | 0.000 | 1.000a |
|  | Yes | 6 (1.2) | 2 (1.0) | 8 (1.2) |  |  |
| antihypertensive drugs | No | 203 (43.5) | 97 (48.0) | 300 (44.8) | 1.181 | 0.310 |
|  | Yes | 264 (56.5) | 105 (52.0) | 369 (55.2) |  |  |
| left pupil shape | Circular | 480 (99.4) | 208 (100) | 688 (99.6) |  | 0.558 |
|  | Non-circular | 3 (0.6) | 0 | 3 (0.4) |  |  |
| right pupil shape | Circular | 484 (100) | 208 (100) | 692 (100) |  |  |
|  | Non-circular | 0 | 0 | 0 |  |  |
| GCS classification | Mild coma | 302 (62.4) | 134 (64.4) | 436 (63.0) | 0.868 | 0.655 |
|  | Moderate coma | 96 (19.8) | 35 (16.8) | 131 (18.9) |  |  |
|  | Severe coma | 86 (17.8) | 39 (18.8) | 125 (18.1) |  |  |
| mRS | 0 | 1 (0.2) | 0 | 1 (0.1) |  | 0.594 |
|  | 1 | 19 (3.9) | 13 (6.3) | 32 (4.6) |  |  |
|  | 2 | 43 (8.9) | 13 (6.3) | 56 (8.1) |  |  |
|  | 3 | 66 (13.6) | 32 (15.4) | 98 (14.2) |  |  |
|  | 4 | 202 (41.7) | 84 (40.4) | 286 (41.3) |  |  |
|  | 5 | 153 (31.6) | 66 (31.7) | 219 (31.6) |  |  |
| subarachnoid hemorrhage | No | 451 (93.2) | 195 (93.8) | 646 (93.4) | 0.076 | 0.869 |
|  | Yes | 33 (6.8) | 13 (6.3) | 46 (6.6) |  |  |
| position of subarachnoid hemorrhage | | |  |  |  |  |
| supratentorial lobes | No | 357 (73.8) | 151 (72.6) | 508 (73.4) | 0.101 | 0.779 |
|  | Yes | 127 (26.2) | 57 (27.4) | 184 (26.6) |  |  |
| frontal lobe | No | 425 (87.8) | 179 (86.1) | 604 (87.3) | 0.402 | 0.535 |
|  | Yes | 59 (12.2) | 29 (13.9) | 88 (12.7) |  |  |
| temporal lobe | No | 421 (87.0) | 179 (86.1) | 600 (86.7) | 0.108 | 0.807 |
|  | Yes | 63 (13.0) | 29 (13.9) | 92 (13.3) |  |  |
| parietal lobe | No | 420 (86.8) | 182 (87.5) | 602 (87.0) | 0.067 | 0.807 |
|  | Yes | 64 (13.2) | 26 (12.5) | 90 (13.0) |  |  |
| occipital lobe | No | 460 (95.0) | 199 (95.7) | 659 (95.2) | 0.128 | 0.847 |
|  | Yes | 24 (5.0) | 9 (4.3) | 33 (4.8) |  |  |
| hemispheres | No | 357 (73.8) | 151 (72.6) | 508 (73.4) |  | 0.369 |
|  | Left | 62 (12.8) | 21 (10.1) | 83 (12.0) |  |  |
|  | Right | 64 (13.2) | 35 (16.8) | 99 (14.3) |  |  |
|  | Both | 1 (0.2) | 1 (0.5) | 2 (0.3) |  |  |
| deep supratentorial hemorrhage | No | 82 (16.9) | 38 (18.3) | 120 (17.3) | 0.179 | 0.743 |
|  | Yes | 402 (83.1) | 170 (81.7) | 572 (82.7) |  |  |
| position of deep supratentorial hemorrhage | |  |  |  |  |  |
| corona radiata | No | 471 (97.3) | 200 (96.2) | 671 (97.0) | 0.665 | 0.469 |
|  | Yes | 13 (2.7) | 8 (3.8) | 21 (3.0) |  |  |
| basal ganglia | No | 151 (31.2) | 69 (33.2) | 220 (31.8) | 0.262 | 0.656 |
|  | Yes | 333 (68.8) | 139 (66.8) | 472 (68.2) |  |  |
| thalamus | No | 359 (74.2) | 148 (71.2) | 507 (73.3) | 0.677 | 0.454 |
|  | Yes | 125 (25.8) | 60 (28.8) | 185 (26.7) |  |  |
| insula | No | 473 (97.7) | 199 (95.7) | 672 (97.1) | 2.187 | 0.214 |
|  | Yes | 11 (2.3) | 9 (4.3) | 20 (2.9) |  |  |
| hemispheres | No | 82 (16.9) | 38 (18.3) | 120 (17.3) |  | 0.693 |
|  | Left | 200 (41.3) | 92 (44.2) | 292 (42.2) |  |  |
|  | Right | 200 (41.3) | 78 (37.5) | 278 (40.2) |  |  |
|  | Both | 2 (0.4) | 0 | 2 (0.3) |  |  |
| intraventricular hemorrhage | No | 311 (64.3) | 130 (62.5) | 441 (63.7) | 0.194 | 0.667 |
|  | Yes | 173 (35.7) | 78 (37.5) | 251 (36.3) |  |  |
| position of intraventricular hemorrhage | | |  |  |  |  |
| lateral ventricular hemorrhage | No | 313 (64.7) | 132 (63.5) | 445 (64.3) | 0.092 | 0.795 |
|  | Yes | 171 (35.3) | 76 (36.5) | 247 (35.7) |  |  |
| hemispheres | No | 313 (64.7) | 132 (63.5) | 445 (64.3) | 3.080 | 0.384 |
|  | Left | 30 (6.2) | 20 (9.6) | 50 (7.2) |  |  |
|  | Right | 29 (6.0) | 14 (6.7) | 43 (6.2) |  |  |
|  | Both | 112 (23.1) | 42 (20.2) | 154 (22.3) |  |  |
| third ventricle | No | 398 (82.2) | 177 (85.1) | 575 (83.1) | 0.850 | 0.378 |
|  | Yes | 86 (17.8) | 31 (14.9) | 117 (16.9) |  |  |
| fourth ventricle | No | 411 (84.9) | 180 (86.5) | 591 (85.4) | 0.307 | 0.639 |
|  | Yes | 73 (15.1) | 28 (13.5) | 101 (14.6) |  |  |
| compression of periventricular tissues | No | 114 (23.6) | 52 (25.0) | 166 (24.0) | 0.167 | 0.698 |
|  | Yes | 370 (76.4) | 156 (75.0) | 526 (76.0) |  |  |
| hydrocephalus | No | 365 (75.4) | 174 (83.7) | 539 (77.9) | 5.737 | 0.021 |
|  | Yes | 119 (24.6) | 34 (16.3) | 153 (22.1) |  |  |
| CT/CTA signs |  |  |  |  |  |  |
| degree of margin irregularity | 1 | 207 (42.9) | 80 (38.5) | 287 (41.6) | 1.965 | 0.745 |
|  | 2 | 143 (29.7) | 63 (30.3) | 206 (29.9) |  |  |
|  | 3 | 60 (12.4) | 28 (13.5) | 88 (12.8) |  |  |
|  | 4 | 25 (5.2) | 15 (7.2) | 40 (5.8) |  |  |
|  | 5 | 47 (9.8) | 22 (10.6) | 69 (10.0) |  |  |
| degree of density heterogeneity | 1 | 285 (59.1) | 131 (63) | 416 (60.3) | 4.937 | 0.295 |
|  | 2 | 138 (28.6) | 55 (26.4) | 193 (28.0) |  |  |
|  | 3 | 39 (8.1) | 15 (7.2) | 54 (7.8) |  |  |
|  | 4 | 12 (2.5) | 1 (0.5) | 13 (1.9) |  |  |
|  | 5 | 8 (1.7) | 6 (2.9) | 14 (2.0) |  |  |
| black hole sign | No | 444 (91.7) | 197 (94.7) | 641 (92.6) | 1.887 | 0.205 |
|  | Yes | 40 (8.3) | 11 (5.3) | 51 (7.4) |  |  |
| island sign | No | 444 (91.7) | 197 (94.7) | 641 (92.6) | 1.887 | 0.205 |
|  | Yes | 40 (8.3) | 11 (5.3) | 51 (7.4) |  |  |
| swirl sign | No | 469 (96.9) | 199 (95.7) | 668 (96.5) | 0.655 | 0.497 |
|  | Yes | 15 (3.1) | 9 (4.3) | 24 (3.5) |  |  |
| fluid-blood level within the hematoma | No | 478 (98.8) | 206 (99.0) | 684 (98.8) | 0.098 | 1.000 |
|  | Yes | 6 (1.2) | 2 (1.0) | 8 (1.2) |  |  |
| else | No | 477 (98.6) | 206 (99.0) | 683 (98.7) | 0.023 | 0.881 |
|  | Yes | 7 (1.4) | 2 (1.0) | 9 (1.3) |  |  |
| vasculopathy | No | 464 (95.9) | 199 (95.7) | 663 (95.8) | 0.014 | 1.000 |
|  | Yes | 20 (4.1) | 9 (4.3) | 29 (4.2) |  |  |

a *P* value was calculated by the continuity-corrected chi-square test; b *P* value was calculated by Log rank test.

**Supplementary Table 3.** Univariate and multivariate analysis of death-related factors in patients with cerebral hemorrhage in the training set.

| Study factors | | survival population (n=356) | death population (n=128) | HR (95% CI) | *P*^a^ | HR (95% CI) | *P*^b^ |
| --- | --- | --- | --- | --- | --- | --- | --- |
| onset-to-hospital interval [hours] | | 12 (6-24) | 10 (6-24) | 0.980 (0.966-0.994) | 0.004 | 1.000 (0.983-1.018) | 0.967 |
| duration of diabetes [years] | | 5 (2-10) | 6 (3.25-10) | 1.030 (0.983-1.080) | 0.213 |  |  |
| duration of hypertension [years] | | 5 (3-10) | 7.5 (4.875-10) | 1.023 (0.996-1.050) | 0.090 |  |  |
| systolic pressure [mmHg] | | 158.32 ± 25.95 | 159.27 ± 31.37 | 1.002 (0.995-1.008) | 0.588 |  |  |
| diastolic pressure [mmHg] | | 92.40 ± 15.42 | 89.40 ± 15.51 | 0.992 (0.981-1.003) | 0.134 |  |  |
| serum creatinine [μmol/L] | | 64.9 (53-79) | 69.6 (58.05-80.25) | 1.000 (0.999-1.002) | 0.579 |  |  |
| blood urea nitrogen [mmol/L] | | 4.86 (3.9-5.81) | 5.26 (4.2-6.37) | 1.001 (0.993-1.009) | 0.799 |  |  |
| uric acid [μmol/L] | | 319.8 (257.2-402.4) | 322.6 (259.7-415.3) | 1.000 (0.999-1.001) | 0.686 |  |  |
| hematoma volume [ml] | | 11.3 (5.1-24.3) | 18.9 (7.9-43.8) | 1.017 (1.012-1.023) | <0.001 | 1.011 (0.998-1.023) | 0.090 |
| edema volume [ml] | | 9.3 (4.1-17.6) | 16.36 (6.96-30.42) | 1.014 (1.008-1.020) | <0.001 | 0.996 (0.984-1.009) | 0.557 |
| age [years] | | 58.15 ± 11.65 | 67.07 ± 11.84 | 1.049 (1.034-1.064) | <0.001 | 1.090 (1.066-1.115) | **<0.001** |
| temperature [℃] | | 36.64 ± 0.39 | 36.70 ± 0.42 | 1.098 (0.741-1.627) | 0.640 |  |  |
| heart rate [times / minute] | | 76.94 ± 14.5 | 82.04 ± 16.1 | 1.015 (1.005-1.026) | 0.004 | 1.009 (0.993-1.025) | 0.269 |
| breath rate [times / minute] | | 20.18 ± 6.73 | 20.48 ± 4.92 | 1.003 (0.981-1.026) | 0.793 |  |  |
| white blood cells [10^9/L] | | 9.48 ± 3.37 | 10.13 ± 4.52 | 1.067 (1.019-1.117) | 0.006 | 1.232 (0.815-1.864) | 0.322 |
| neutrophils [10^9/L] | | 7.67 ± 5.02 | 8.87 ± 8.44 | 1.020 (0.999-1.041) | 0.059 | 0.850 (0.556-1.302) | 0.456 |
| lymphocytes [10^9/L] | | 1.39 ± 1.34 | 1.15 ± 0.77 | 0.663 (0.474-0.926) | 0.016 | 0.951 (0.646-1.401) | 0.801 |
| red blood cells [10^12/L] | | 4.65 ± 0.57 | 4.43 ± 0.63 | 0.674 (0.523-0.869) | 0.002 | 0.885 (0.575-1.364) | 0.580 |
| hemoglobin [g/L] | | 141.88 ± 17.04 | 136.57 ± 18.67 | 0.993 (0.986-1.001) | 0.073 |  |  |
| hematocrit [L/L] | | 1.15 ± 11.52 | 0.40 ± 0.05 | 0.011 (0.000-0.616) | 0.028 | 0.969 (0.515-1.821) | 0.922 |
| platelets [10^9/L] | | 212.67 ± 66.76 | 200.85 ± 73.61 | 0.996 (0.993-0.998) | 0.001 | 0.997 (0.994-1.001) | 0.161 |
| Fasting blood glucose [mol/L] | | 6.85 ± 2.80 | 8.13 ± 3.82 | 1.088 (1.040-1.139) | <0.001 | 1.073 (0.998-1.155) | 0.058 |
| K+ [mmol/L] | | 3.83 ± 0.42 | 3.83 ± 0.48 | 1.015 (0.662-1.557) | 0.944 |  |  |
| Na+ [mmol/L] | | 140.27 ± 3.74 | 139.88 ± 4.68 | 0.992 (0.948-1.038) | 0.720 |  |  |
| Cl- [mmol/L] | | 100.98 ± 16.81 | 100.12 ± 16.73 | 0.994 (0.982-1.006) | 0.317 |  |  |
| anion gap [mmol/L] | | 12.24 ± 5.14 | 13.38 ± 5.07 | 1.012 (0.993-1.031) | 0.219 |  |  |
| prothrombin time [seconds] | | 11.44 ± 1.79 | 12.05 ± 2.89 | 1.061 (1.019-1.106) | 0.004 | 0.906 (0.784-1.048) | 0.183 |
| PT-INR |  | 0.95 ± 0.27 | 1.03 ± 0.28 | 2.017 (1.325-3.069) | 0.001 | 4.304 (0.986-18.794) | 0.052 |
| fibrinogen [g/L] | | 2.71 ± 0.76 | 2.90 ± 0.99 | 1.107 (0.888-1.381) | 0.367 |  |  |
| thrombin time [seconds] | | 17.51 ± 7.23 | 19.03 ± 13.5 | 1.007 (0.996-1.018) | 0.209 |  |  |
| sex | Female | 119 (33.4) | 30 (23.4) | 1 | -- | 1 | -- |
|  | Male | 237 (66.6) | 98 (76.6) | 1.601 (1.063-2.412) | 0.024 | 1.671 (0.990-2.820) | 0.055 |
| consciousness disorder | No | 224 (62.9) | 45 (35.2) | 1 | -- | 1 | -- |
|  | Yes | 132 (37.1) | 83 (64.8) | 2.775 (1.929-3.993) | <0.001 | 1.043 (0.571-1.906) | 0.890 |
| epilepsy | No | 345 (96.9) | 122 (95.3) | 1 | -- | 1 | -- |
|  | Yes | 11 (3.1) | 6 (4.7) | 1.513 (0.666-3.438) | 0.323 |  |  |
| headache | No | 227 (63.8) | 97 (75.8) | 1 | -- | 1 | -- |
|  | Yes | 129 (36.2) | 31 (24.2) | 0.569 (0.380-0.853) | 0.006 | 0.866 (0.518-1.445) | 0.581 |
| neurological dysfunction | No | 57 (16.0) | 33 (25.8) | 1 | -- | 1 | -- |
|  | Yes | 299 (84.0) | 95 (74.2) | 0.585 (0.394-0.870) | 0.008 | 0.682 (0.403-1.156) | 0.155 |
| smoke | No | 264 (74.2) | 92 (71.9) | 1 | -- |  |  |
|  | Yes | 92 (25.8) | 36 (28.1) | 1.216 (0.827-1.789) | 0.321 |  |  |
| drink | No | 261 (73.3) | 101 (78.9) | 1 | -- |  |  |
|  | Yes | 95 (26.7) | 27 (21.1) | 0.852 (0.557-1.304) | 0.461 |  |  |
| medical history | |  |  |  |  |  |  |
| diabetes | No | 322 (90.4) | 107 (83.6) | 1 | -- | 1 | -- |
|  | Yes | 34 (9.6) | 21 (16.4) | 1.737 (1.087-2.775) | 0.021 | 1.116 (0.577-2.157) | 0.744 |
| control of diabetes | No | 322 (90.4) | 107 (83.6) | 1 | -- |  |  |
|  | Good | 2 (0.6) | 2 (1.6) | 2.982 (0.735-12.105) | 0.126 |  |  |
|  | Poor | 3 (0.8) | 4 (3.1) | 2.932 (1.079-7.968) | 0.035 |  |  |
|  | Uncontrolled | 4 (1.1) | 0 | -- | 0.960 |  |  |
|  | Unknown | 25 (7.0) | 15 (11.7) | 1.700 (0.989-2.923) | 0.055 |  |  |
| hypertension | No | 39 (11.0) | 17 (13.3) | 1 | -- | 1 | -- |
|  | Present illness | 254 (71.3) | 98 (76.6) | 0.770 (0.460-1.290) | 0.321 | 0.664 (0.337-1.310) | 0.238 |
|  | Newly diagnosed | 63 (17.7) | 13 (10.2) | 0.470 (0.228-0.968) | 0.040 | 0.366 (0.142-0.944) | 0.038 |
| control of hypertension | No | 102 (28.7) | 30 (23.4) | 1 | -- |  |  |
|  | Good | 15 (4.2) | 5 (3.9) | 1.132 (0.439-2.918) | 0.798 |  |  |
|  | Poor | 24 (6.7) | 9 (7.0) | 1.164 (0.553-2.453) | 0.689 |  |  |
|  | Uncontrolled | 26 (7.3) | 9 (7.0) | 1.038 (0.492-2.189) | 0.922 |  |  |
|  | Unknown | 189 (53.1) | 75 (58.6) | 1.161 (0.760-1.774) | 0.491 |  |  |
| dyslipidemia | No | 353 (99.2) | 128 (100) | 1 | -- |  |  |
|  | Yes | 3 (0.8) | 0 | -- | 0.569a |  |  |
| heart diseases | No | 342 (96.1) | 119 (93.0) | 1 | -- |  |  |
|  | Yes | 14 (3.9) | 9 (7.0) | 1.345 (0.682-2.652) | 0.392 |  |  |
| hematological diseases | No | 355 (99.7) | 127 (99.2) | 1 | -- |  |  |
|  | Yes | 1 (0.3) | 1 (0.8) | 1.928 (0.269-13.82) | 0.514 |  |  |
| cerebrovascular diseases | No | 335 (94.1) | 116 (90.6) | 1 | -- |  |  |
|  | Yes | 21 (5.9) | 12 (9.4) | 1.350 (0.745-2.447) | 0.322 |  |  |
| dementia | No | 354 (99.7) | 127 (99.2) | 1 | -- |  |  |
|  | Yes | 1 (0.3) | 1 (0.8) | 1.082 (0.151-7.777) | 0.938 |  |  |
| liver diseases | No | 340 (95.5) | 120 (93.8) | 1 | -- |  |  |
|  | Yes | 16 (4.5) | 8 (6.3) | 1.306 (0.638-2.672) | 0.465 |  |  |
| premobid mRS | 0 | 320 (89.9) | 105 (82.0) | 1 | -- |  |  |
|  | 1 | 36 (10.1) | 23 (18.0) | 1.534 (0.975-2.412) | 0.064 |  |  |
| Family history | |  |  |  |  |  |  |
| hypertension | No | 326 (91.8) | 122 (95.3) | 1 | -- |  |  |
|  | Yes | 29 (8.2) | 6 (4.7) | 0.565 (0.249-1.284) | 0.173 |  |  |
| hyperlipidemia | No | 355 (100) | 128 (100) |  |  |  |  |
|  | Yes | 0 | 0 |  |  |  |  |
| diabetes | No | 350 (98.6) | 126 (98.4) | 1 | -- |  |  |
|  | Yes | 5 (1.4) | 2 (1.6) | 1.594 (0.393-6.469) | 0.514 |  |  |
| stroke | No | 347 (97.7) | 125 (97.7) | 1 | -- |  |  |
|  | Yes | 8 (2.3) | 3 (2.3) | 0.942 (0.300-2.961) | 0.918 |  |  |
| coronary heart disease | No | 355 (100) | 128 (100) |  |  |  |  |
|  | Yes | 0 | 0 |  |  |  |  |
| Medication history | |  |  |  |  |  |  |
| antiplatelet drugs | No | 347 (97.5) | 125 (98.4) | 1 | -- |  |  |
|  | Yes | 9 (2.5) | 2 (1.6) | 0.666 (0.165-2.695) | 0.569 |  |  |
| anticoagulant drugs | No | 353 (99.2) | 125 (97.7) | 1 | -- |  |  |
|  | Yes | 3 (0.8) | 3 (2.3) | 2.478 (0.787-7.798) | 0.121 |  |  |
| antihypertensive drugs | No | 156 (45.0) | 47 (39.2) | 1 | -- |  |  |
|  | Yes | 191 (55.0) | 73 (60.8) | 1.174 (0.814-1.694) | 0.390 |  |  |
| left pupil shape | Circular | 353 (99.2) | 127 (100) | -- | 0.570a |  |  |
|  | Non-circular | 3 (0.8) | 0 |  |  |  |  |
| right pupil shape | Circular | 356 (100) | 128 (100) | -- | -- |  |  |
|  | Non-circular | 0 | 0 |  |  |  |  |
| GCS classification | Mild coma | 253 (71.1) | 49 (38.3) | 2.237 (1.827-2.738) | <0.001 | 1.721 (1.161-2.551) | **0.007** |
|  | Moderate coma | 63 (17.7) | 33 (25.8) |  |  |  |  |
|  | Severe coma | 40 (11.2) | 46 (35.9) |  |  |  |  |
| mRS | 0 | 1 (0.3) | 0 | 1.477 (1.212-1.800) | <0.001 | 1.145 (0.866-1.512) | 0.342 |
|  | 1 | 13 (3.7) | 6 (4.7) |  |  |  |  |
|  | 2 | 37 (10.4) | 6 (4.7) |  |  |  |  |
|  | 3 | 52 (14.6) | 14 (10.9) |  |  |  |  |
|  | 4 | 168 (47.2) | 34 (26.6) |  |  |  |  |
|  | 5 | 85 (23.9) | 68 (53.1) |  |  |  |  |
| subarachnoid hemorrhage | No | 333 (93.5) | 118 (92.2) | 1 | -- |  |  |
|  | Yes | 23 (6.5) | 10 (7.8) | 1.271 (0.666-2.425) | 0.467 |  |  |
| position of subarachnoid hemorrhage | | |  |  |  |  |  |
| supratentorial lobes | No | 272 (76.4) | 85 (66.4) | 1 | -- |  |  |
|  | Yes | 84 (23.6) | 43 (33.6) | 1.444 (1.00-2.085) | 0.05 |  |  |
| frontal lobe | No | 321 (90.2) | 104 (81.3) | 1 | -- | 1 | -- |
|  | Yes | 35 (9.8) | 24 (18.8) | 2.026 (1.299-3.162) | 0.002 | 0.791 (0.338-1.855) | 0.590 |
| temporal lobe | No | 315 (88.5) | 106 (82.8) | 1 | -- |  |  |
|  | Yes | 41 (11.5) | 22 (17.2) | 1.341 (0.847-2.124) | 0.211 |  |  |
| parietal lobe | No | 308 (86.5) | 112 (87.5) | 1 | -- |  |  |
|  | Yes | 48 (13.5) | 16 (12.5) | 0.850 (0.503-1.437) | 0.544 |  |  |
| occipital lobe | No | 341 (95.8) | 119 (93.0) | 1 | -- |  |  |
|  | Yes | 15 (4.2) | 9 (7.0) | 1.283 (0.649-2.533) | 0.473 |  |  |
| hemispheres | No | 272 (76.4) | 85 (66.4) | 1 | -- | 1 | -- |
|  | Left | 50 (14.0) | 12 (9.4) | 0.789 (0.431-1.445) | 0.443 | 0.403 (0.182-0.894) | 0.025 |
|  | Right | 33 (9.3) | 31 (24.2) | 2.161 (1.431-3.261) | <0.001 | 1.974 (0.897-4.346) | 0.091 |
|  | Both | 1 (0.3) | 0 | -- | 0.961 | -- | 0.967 |
| deep supratentorial hemorrhage | No | 58 (16.3) | 24 (18.8) | 1 | -- |  |  |
|  | Yes | 298 (83.7) | 104 (81.3) | 0.866 (0.555-1.350) | 0.526 |  |  |
| position of deep supratentorial hemorrhage | | |  |  |  |  |  |
| corona radiata | No | 349 (98.0) | 122 (95.3) | 1 | -- |  |  |
|  | Yes | 7 (2.0) | 6 (4.7) | 1.472 (0.647-3.349) | 0.357 |  |  |
| basal ganglia | No | 107 (30.1) | 44 (34.4) | 1 | -- |  |  |
|  | Yes | 249 (69.9) | 84 (65.6) | 0.908 (0.630-1.309) | 0.604 |  |  |
| thalamus | No | 272 (76.4) | 87 (68.0) | 1 | -- |  |  |
|  | Yes | 84 (23.6) | 41 (32.0) | 1.421 (0.980-2.061) | 0.064 |  |  |
| insula | No | 348 (97.8) | 125 (97.7) | 1 | -- |  |  |
|  | Yes | 8 (2.2) | 3 (2.3) | 1.078 (0.343-3.388) | 0.898 |  |  |
| hemispheres | No | 58 (16.3) | 24 (18.8) | 1 | -- |  |  |
|  | Left | 154 (43.3) | 46 (35.9) | 0.761 (0.465-1.247) | 0.279 |  |  |
|  | Right | 143 (40.2) | 57 (44.5) | 0.967 (0.600-1.559) | 0.891 |  |  |
|  | Both | 1 (0.3) | 1 (0.8) | 1.406 (0.190-10.404) | 0.739 |  |  |
| intraventricular hemorrhage† | No | 248 (69.7) | 63 (49.2) | 1 | -- |  |  |
|  | Yes | 108 (30.3) | 65 (50.8) | 2.239 (1.579-3.173) | <0.001 |  |  |
| position of intraventricular hemorrhage | | |  |  |  |  |  |
| lateral ventricular hemorrhage† | No | 250 (70.2) | 63 (49.2) | 1 | -- |  |  |
|  | Yes | 106 (29.8) | 65 (50.8) | 2.304 (1.625-3.267) | <0.001 |  |  |
| hemispheres | No | 250 (70.2) | 63 (49.2) | 1 | -- | 1 | -- |
|  | Left | 24 (6.7) | 6 (4.7) | 1.207 (0.521-2.793) | 0.661 | 1.603 (0.596-4.312) | 0.350 |
|  | Right | 20 (5.6) | 9 (7.0) | 1.706 (0.848-3.434) | 0.134 | 0.682 (0.236-1.973) | 0.480 |
|  | Both | 62 (17.4) | 50 (39.1) | 2.784 (1.915-4.048) | <0.001 | 0.879 (0.455-1.699) | 0.702 |
| third ventricle | No | 307 (86.2) | 91 (71.1) | 1 | -- | 1 | -- |
|  | Yes | 49 (13.8) | 37 (28.9) | 2.325 (1.584-3.414) | <0.001 | 1.566 (0.551-4.447) | 0.400 |
| fourth ventricle | No | 313 (87.9) | 98 (76.6) | 1 | -- | 1 | -- |
|  | Yes | 43 (12.1) | 30 (23.4) | 2.179 (1.445-3.284) | <0.001 | 0.731 (0.258-2.073) | 0.556 |
| compression of periventricular tissues | No | 93 (26.1) | 21 (16.4) | 1 | -- |  |  |
|  | Yes | 263 (73.9) | 107 (83.6) | 1.481 (0.926-2.367) | 0.101 |  |  |
| hydrocephalus | No | 296 (83.1) | 69 (53.9) | 1 | -- | 1 | -- |
|  | Yes | 60 (16.9) | 59 (46.1) | 3.092 (2.183-4.378) | <0.001 | 2.418 (1.449-4.033) | **0.001** |
| CT/CTA signs | |  |  |  |  |  |  |
| degree of margin irregularity | 1 | 161 (45.4) | 46 (36.2) | 1.215 (1.073-1.374) | 0.002 | 0.917 (0.764-1.101) | 0.353 |
|  | 2 | 109 (30.7) | 34 (26.8) |  |  |  |  |
|  | 3 | 43 (12.1) | 17 (13.4) |  |  |  |  |
|  | 4 | 18 (5.1) | 7 (5.5) |  |  |  |  |
|  | 5 | 24 (6.8) | 23 (18.1) |  |  |  |  |
| degree of density heterogeneity | 1 | 223 (62.8) | 62 (48.8) | 1.363 (1.154-1.608) | <0.001 | 1.079 (0.811-1.435) | 0.603 |
|  | 2 | 100 (28.2) | 38 (29.9) |  |  |  |  |
|  | 3 | 21 (5.9) | 18 (14.2) |  |  |  |  |
|  | 4 | 7 (2.0) | 5 (3.9) |  |  |  |  |
|  | 5 | 4 (1.1) | 4 (3.1) |  |  |  |  |
| black hole sign | No | 332 (93.3) | 112 (87.5) | 1 | -- | 1 | -- |
|  | Yes | 24 (6.7) | 16 (12.5) | 1.856 (1.097-3.138) | 0.021 | 0.665 (0.317-1.397) | 0.281 |
| island sign | No | 327 (91.9) | 117 (91.4) | 1 | -- |  |  |
|  | Yes | 29 (8.1) | 11 (8.6) | 1.048 (0.565-1.946) | 0.881 |  |  |
| swirl sign | No | 347 (97.5) | 122 (95.3) | 1 | -- |  |  |
|  | Yes | 9 (2.5) | 6 (4.7) | 1.839 (0.809-4.181) | 0.146 |  |  |
| fluid-blood level within the hematoma | No | 353 (99.2) | 125 (97.7) | 1 | -- |  |  |
|  | Yes | 3 (0.8) | 3 (2.3) | 2.333 (0.740-7.354) | 0.148 |  |  |
| else | No | 349 (98.0) | 128 (100) | 1 | -- |  |  |
|  | Yes | 7 (2.0) | 0 | 0.048 (0.000-21.428) | 0.330 |  |  |
| vasculopathy | No | 342 (96.1) | 118 (95.2) | 1 | -- |  |  |
|  | Yes | 14 (3.9) | 6 (4.8) | 1.307 (0.575-2.968) | 0.523 |  |  |

^a^ HR value and *P* value was calculated by univariate Cox Proportional Hazard Model; ^b^ HR value and *P* value was calculated by multivariate cox proportional hazard model adjusted by meaningful variables identified in univariate Cox Proportional Hazard Model.

† The variable was not included in the multivariate cox proportional hazard model due to collinearity.

HR, Hazard ratio.


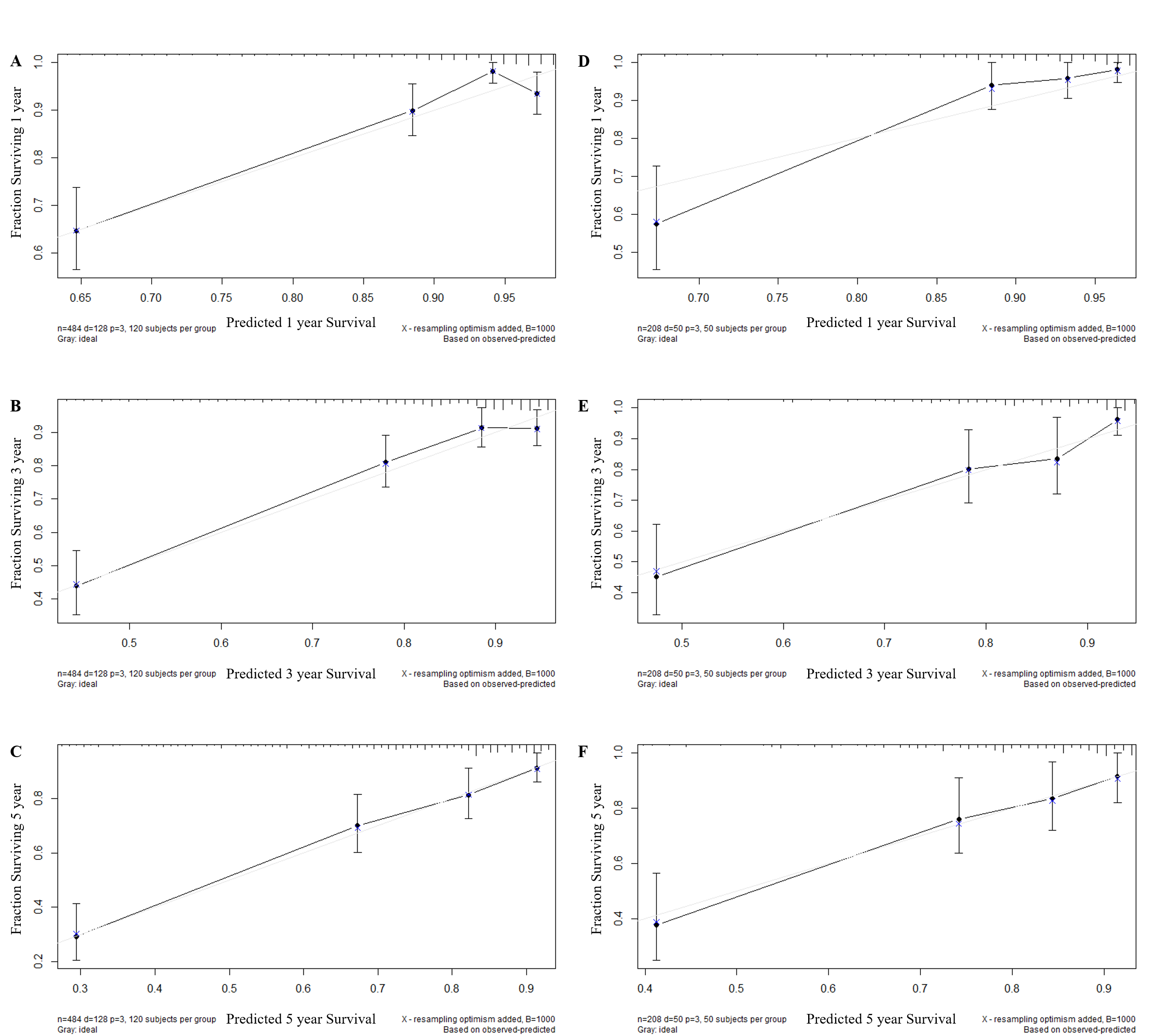


**Supplementary Figure 1.** Calibration curve of predictive model. A. 1-year calibration curve of the training cohort; B. 3-years calibration curve of the training cohort; C. 5-years calibration curve of the training cohort; D. 1-year calibration curve of the validation cohort; E. 3-years calibration curve of the validation cohort; F. 5-years calibration curve of the validation cohort. Survival analysis was performed with Survival R package version 3.3-1 (Threneau 2022). Regression modeling strategies were performed with Rms version 6.3-0 (Harrell 2022).


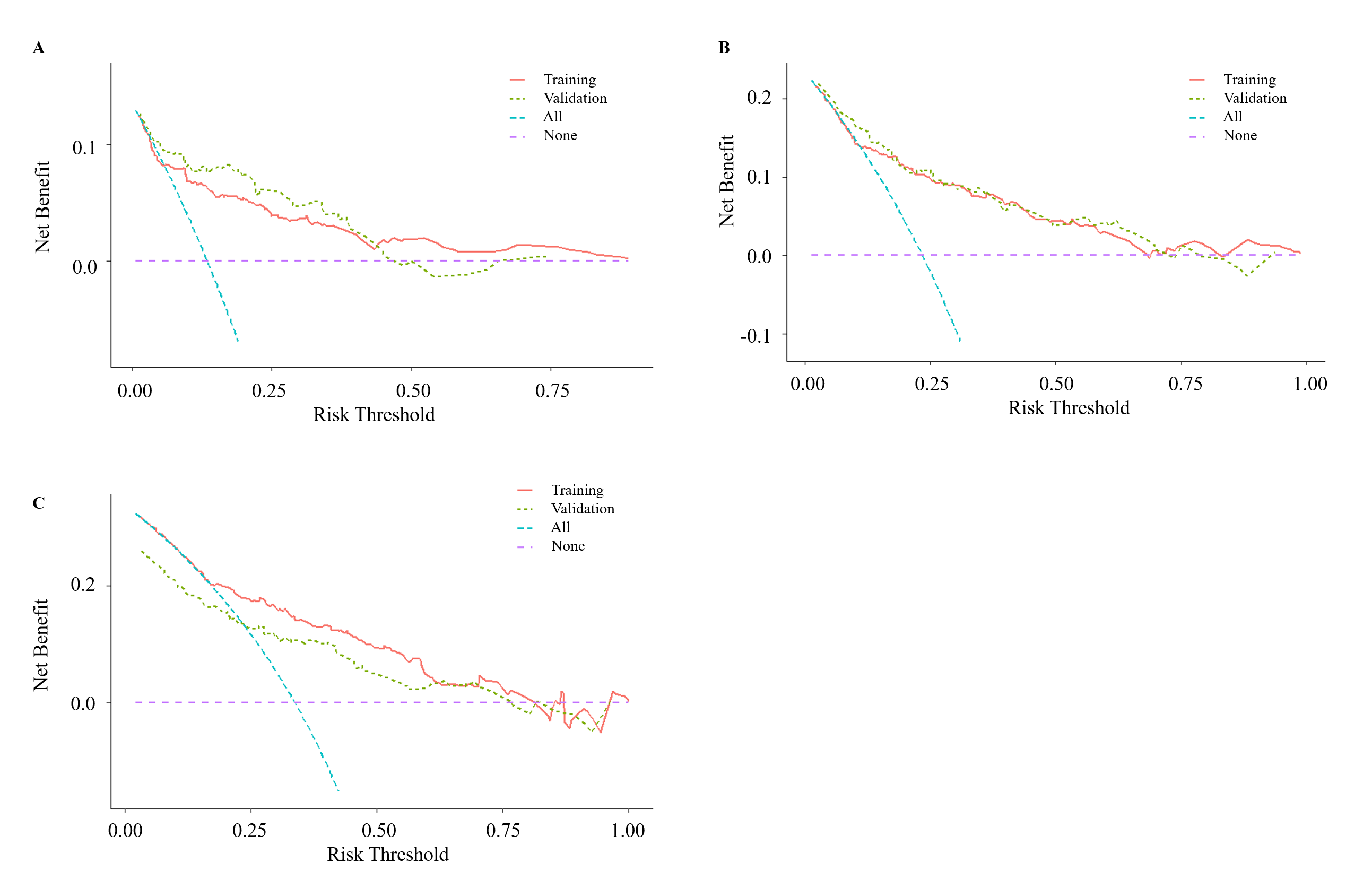


**Supplementary Figure 2.** Decision curve of predictive model. A. 1-year decision curve; B. 3-years decision curve; C. 5-years decision curve. The red line represents the training cohort; The green line represents the validation cohort. The blue and purple lines represent the two extreme cases, with blue representing all subjects as patients and purple representing all subjects as non-patients. The coincidence degree of the two lines indicates the coincidence degree of the prediction effects of the two data sets. Decision curve was performed with ggDCA R version 1.1 (Harrell 2022) and Foreign R package version 0.8-82 (R Core Team 2022).
